# Machine learning based prediction models for cardiovascular disease risk using electronic health records data: systematic review and meta-analysis

**DOI:** 10.1093/ehjdh/ztae080

**Published:** 2024-10-27

**Authors:** Tianyi Liu, Andrew Krentz, Lei Lu, Vasa Curcin

**Affiliations:** School of Life Course & Population Sciences, King's College London, SE1 1UL London, UK; School of Life Course & Population Sciences, King's College London, SE1 1UL London, UK; Metadvice, 45 Pall Mall, St. James’s SW1Y 5JG London, UK; School of Life Course & Population Sciences, King's College London, SE1 1UL London, UK; School of Life Course & Population Sciences, King's College London, SE1 1UL London, UK

**Keywords:** Cardiovascular diseases, Machine learning, Electronic health records, Risk prediction, Primary prevention

## Abstract

Cardiovascular disease (CVD) remains a major cause of mortality in the UK, prompting the need for improved risk predictive models for primary prevention. Machine learning (ML) models utilizing electronic health records (EHRs) offer potential enhancements over traditional risk scores like QRISK3 and ASCVD. To systematically evaluate and compare the efficacy of ML models against conventional CVD risk prediction algorithms using EHR data for medium to long-term (5–10 years) CVD risk prediction. A systematic review and random-effect meta-analysis were conducted according to preferred reporting items for systematic reviews and meta-analyses guidelines, assessing studies from 2010 to 2024. We retrieved 32 ML models and 26 conventional statistical models from 20 selected studies, focusing on performance metrics such as area under the curve (AUC) and heterogeneity across models. ML models, particularly random forest and deep learning, demonstrated superior performance, with the highest recorded pooled AUCs of 0.865 (95% CI: 0.812–0.917) and 0.847 (95% CI: 0.766–0.927), respectively. These significantly outperformed the conventional risk score of 0.765 (95% CI: 0.734–0.796). However, significant heterogeneity (I² > 99%) and potential publication bias were noted across the studies. While ML models show enhanced calibration for CVD risk, substantial variability and methodological concerns limit their current clinical applicability. Future research should address these issues by enhancing methodological transparency and standardization to improve the reliability and utility of these models in clinical settings. This study highlights the advanced capabilities of ML models in CVD risk prediction and emphasizes the need for rigorous validation to facilitate their integration into clinical practice.

## Graphical Abstract

**Graphical Abstract:**
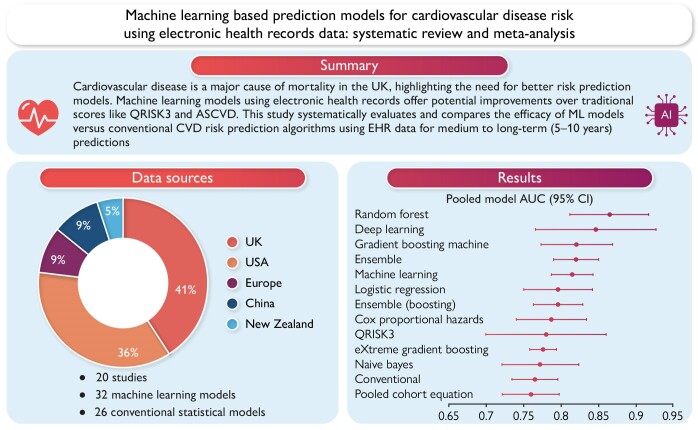

## Introduction

Cardiovascular disease (CVD) is a significant public health issue in the UK, affecting about 7.6 million people as of 2024.^1^ In 2022, CVD was responsible for approximately 174 884 deaths, accounting for 26% of all fatalities.^1^ Non-modifiable risk factors such as age, gender, ethnicity, and family history, as well as modifiable risk factors like smoking, alcohol consumption, poor diet, and physical inactivity, play crucial roles in CVD development.^2^ Additionally, conditions like hypertension, obesity, and high cholesterol further contribute to the disease burden.^2,3^ Regular screenings and timely medical interventions can help mitigate CVD onset and severity, improve health outcomes, and reduce the overall burden.^4^ In the UK, individuals aged 40 and over are offered CVD risk assessments by their general practitioner every 5 years.^2^

Several CVD prediction algorithms estimate 10-year CVD risk based on known factors and utilized in primary care. The QRISK3^5^ in the UK and ASCVD^6^ (atherosclerotic CVD) in the USA are the most widely used and have been validated for primary prevention through decision on statin prescriptions and other therapies. These algorithms are endorsed by national guidelines like NICE^2^ (National Institute for Health and Care Excellence) in the UK and ACC/AHA^7^ (American College of Cardiology, American Heart Association) in the USA, significantly impacting clinical practice. *Table 1* shows the CVD risk scores currently used in clinical practice, their corresponding guidelines, and conventional models used. Efforts to enhance these algorithms include integrating new predictor variables^8^ and regular external validation or recalibration across different populations.^9,10^

**Table 1:** Overview of global cardiovascular risk score models and guidelines

Country/region	Guidelines for recommend	CVD risk score	Model used
UK	National Institute for Health and Care Excellence (NICE)	QRISK	Cox proportional hazards
Scotland	Scottish Intercollegiate Guidelines Network (SIGN)	ASSIGN	Cox proportional hazards
USA	American College of Cardiology		
	American Heart Association (AHA/ACC)	Framingham Risk Score	Cox proportional hazards
USA	American College of Cardiology		
	American Heart Association (AHA/ACC)	ASCVD Risk Estimator Plus	Pooled cohort equations
Europe	European Society of Cardiology (ESC)	SCORE	Logistic regression
China	Guideline on the Assessment and Management of Cardiovascular Risk in China	China-PAR	Cox proportional hazards
Japan	Japan Atherosclerosis Society (JAS)	JALS Score	Cox proportional hazards
Singapore	Agency for Care Effectiveness (ACE)	Singapore Cardiovascular Risk Prediction Score	Cox proportional hazards
Australia	National Vascular Disease Prevention Alliance (NVPDA)	Australian Absolute CVD Risk Calculator	Cox proportional hazards
New Zealand	Ministry of Health	NZ Primary Prevention Equation	Cox proportional hazards
South Africa	Heart and Stroke Foundation S.A.	South African Risk Charts	Logistic Regression
United Arab Emirates	Emirates Cardiac Society	UAE Heart Risk Calculator	Cox proportional hazards
Global	World Health Organization	Cardiovascular Risk Charts (WHO/ISH)	Cox proportional hazards
Global	World Health Organization	Globorisk	Cox proportional hazards

QRISK and ASCVD were established using Cox proportional-hazard models (Cox) and pooled cohort equations (PCE), respectively.^5,6^ Despite their validation, some researchers suggest these algorithms may misestimate risk for certain subpopulations^11,12^ or yield controversial outcomes for specific predictors.^13^

Machine learning (ML) and deep learning (DL) have revolutionized CVD risk research,^14^ using sophisticated algorithms to identify complex patterns in diverse datasets that conventional methods might eclude^15^ These approaches focus on discerning concealed correlations within clinical data, encompassing both the structured and unstructured facets of electronic health records (EHRs). DL, particularly, advances this endeavour by utilizing artificial neural networks (NN) that emulate human cognitive functions to extract data representations, thus enabling more refined risk stratification for CVD patients. Despite challenges like model interpretability and overfitting, ML and DL are advancing personalized CVD risk evaluation and management.^16^

EHRs have precipitated a paradigm shift in the prognostication of CVD, serving as a repository of comprehensive patient data. These digitized records compile an array of information, including medical histories, demographic data, and laboratory results, which are invaluable for predicting disease trajectories and prognosticating patient outcomes.^17^ Researchers have developed sophisticated ML models using EHRs that outperform conventional algorithms in discrimination and calibration, thereby shedding new light on CVD management.^18^ EHRs also facilitate the assimilation of heterogeneous data modalities, such as genetic profiles and medical imaging, thereby broadening the scope of cardiovascular research.^19^ The structured nature of EHRs, buttressed by uniform clinical terminologies, promotes system interoperability and underpins the development of predictive models that account for patient diversity.^20^ The expansion of EHR-based studies is creating a robust framework for CVD prediction, characterized by extensive, generalizable patient cohorts and rich, multi-faceted datasets.

Recent studies indicate that ML/DL techniques offer superior long-term CVD risk prediction and specific events like myocardial infarction^21^ (MI), ischaemic stroke,^22^ and heart failure^23^ (HF). These models outperform traditional algorithms like QRISK and ASCVD in discrimination and calibration, better accommodating patient heterogeneity and comorbidities.^15^ The increasing use of EHR datasets for model development and validation highlights EHRs’ superiority over traditional cohorts for ML model development.

### Rationale and objectives

The primary prevention risk prediction models for CVD face a gap in thorough evaluation, especially for those using ML techniques and EHRs. While conventional risk scores are practically applied, the claimed superior performance of new ML algorithms is unconfirmed due to a lack of in-depth comparative analyses and external validation. Comparisons among ML models are often restricted to specific datasets and lack practical implementation,^24^ with sparse discussion on methodologies. EHR-based models have the potential to surpass traditional cohort-based models but are underutilized. Our specific goals are:

1. To extract and document the characteristics of selected eligible models employed in previous literature, examining the degree of consistency or variation across different model settings.
2. To evaluate the risk of bias in each study, considering factors both internal and external to ensure the validity of our findings.
3. To synthesize, summarize, and compare the overall efficacy and performance of the selected models, focusing on discrimination measures such as the C-statistic.
4. To assess heterogeneity among individual studies, identifying potential sources of variation and assessing their impact on the review's overall findings.

This systematic review consolidates evidence on ML-based risk prediction models using EHRs for CVD primary prevention, aiming to assess and compare the performance of various algorithms, thereby informing clinical practice and guiding future research in the evolving field of CVD risk prediction.

## Methods

Our systematic review is conducted in accordance with the guidelines outlined in the preferred reporting items for systematic reviews and meta-analyses^25^ (PRISMA).

### Identification of studies

The search for relevant studies was conducted comprehensively in March 2024. Initial searches were performed on electronic databases, including PubMed/MEDLINE and Embase, spanning the years 2010–24. A combination of Medical Subject Headings (MeSH) terms and free text related to ‘CVD’, ‘ML’, ‘EHR’, and ‘risk assessment/factors’ was employed to identify studies published since 1 January 2010.

We refined our search by applying filters to include studies conducted on humans, publications that have been peer-reviewed, those written in English, and those for which full texts were available. A comprehensive search log and the strategies used are provided in the Appendix for transparency and reproducibility. To supplement the primary search, we performed a backwards search in the reference lists of selected studies using ISI Web of Science.

### Selection of studies

We compiled all search results and materials from various sources and eliminated any duplicate papers using Rayyan,^26^ an online application specifically designed for preliminary screening in systematic reviews.

Initially, we independently screened the titles and abstracts to discard any irrelevant papers or information. Then, we assessed the full text of the remaining papers to identify those that could potentially be included in the review. This process facilitated the decision-making on which studies were most suitable for inclusion. Any disagreements that arose during this process were resolved through consensus.

For effective management of our bibliography, we utilized Zotero,^27^ a widely used reference management software.

### Eligibility criteria

*Table 2* provides a detailed breakdown of the inclusion and exclusion criteria applied in this systematic review. The main emphasis is the investigation of ML-based risk models that predict medium- or long-term (e.g. 5–15 years, lifetime) CVD outcomes for primary prevention. These models should be primarily built on structured patient-level EHR data or partially integrated EHR data, while also incorporating cohort studies such as the UK Biobank. The models should employ multiple variables or predictors, without solely relying on biomarkers, image, or genetic data. The outcomes of interest could encompass one or multiple CVD conditions with definition using controlled terminology such as International Classification of Diseases (ICD). Studies that report on the validation, modification, updates, and comparisons with conventional statistical models are also included, provided they present some degree of performance metrics, including calibration and discrimination.

**Table 2:** Inclusion and exclusion criteria for selected studies

	Inclusion criteria	Exclusion criteria
Publication type	Original studies which report the development and/or validation, and/or comparison of models	Methodology studies, editorial comments, research protocols, review or systematic review.
Settings	Outpatients/GP.	Inpatients/hospitalizations, ED, or remote monitoring at home.
Models and tasks	Multivariable ML/DL models for long-term individual risk prediction.	Studies that only report conventional statistical methods, feature selection, and ML models for embedding, NLP, and subtype definition/clustering.
Populations	Adults (18 years of age and older), asymptomatic general population.	Patients with prior CVD or CV symptoms. Specific sub-populations including particular ethnicities, genders, age groups, or patients with specific diseases.
Data type	Structure individual data derived or integrated from electronic health (medical) records	The data only contain ECGs, echocardiograms, ultrasounds, DNA sequences, and other imaging data.
CVD outcomes	CHD, stroke/TIA, or heart failure	In-hospital CVD outcomes including survival/mortality after surgery, (re)admission, and length of stay.
Filter applied	Published after January 2010, publication in English, human studies, full-text available, peer-reviewed literature.	

GP, general practice; ED, emergency department; ML/DL, machine/deep learning; CVD, cardiovascular diseases; ECG, electrocardiogram; CHD, coronary heart disease; TIA, transient ischaemic attack.

Exclusion criteria for this review include non-English and non-human studies, as well as review articles. Models that primarily predict cardiac complications, length of stay, readmissions, or future procedure therapy during hospitalization, after surgery, or in emergency departments (ED) are excluded.

Importantly, our focus is on models developed for asymptomatic individuals with no prior cardiovascular events, i.e. primary prevention, as opposed to models that predict CVD risk in patients with specific high-risk conditions such as diabetes, chronic kidney disease (CKD), post-MI, or within particular demographic groups such as gender, ethnicity, or elderly people.

### Data items collection and extraction

We utilized Microsoft Excel^28^ to independently extract data items from the studies that met the inclusion criteria. Cross-checking of data extraction was performed, and any conflicts were resolved by consensus. Extraction checklist tools were developed and modified based on consensus, drawing upon established frameworks including TRIPOD-AI^29^ (an extension of the Transparent Reporting of a multivariable prediction model for Individual Prognosis or Diagnosis statement for AI) and CHARMS^30^ (Critical Appraisal and Data Extraction for Systematic Reviews of Prediction Modelling Studies).

From each study, we extracted information spanning nine domains, totalling 39 key items. This included references, data sources, participants, CVD outcomes to be predicted, predictors/features, handling of missing data, model development, model performance, and model evaluation, as shown in *Table 3*. The complete data extraction is available in Supplementary material online, files  *S1* for reference and further analysis.

**Table 3:** Key items for retrieving from selected studies

Domain	Key items
1. Reference	1.1. First Author
	1.2. Publication year
	1.3. Published journal
	1.4. Country/Region
	1.5. Objective of the study
2. Data source	2.1. Source of Data
	2.2. Data period
	2.3. Follow-up duration
	2.4. Sample size
3. Participants	3.1. Inclusion and exclusion criteria
	3.2. Settings
	3.3. Number of centres
4. CVD outcomes	4.1. Clinical outcome
	4.2. Was the outcome distribution unbalanced?
	4.3. Number of outcomes/events
5. Features	5.1. Feature used before feature selection reported
	5.2. Feature used for algorithms reported
	5.3. Number of Predictors/Features
	5.4. Type of predictors included
6. Missing data	6.1. Were there any missing values?
	6.2. Were any variables removed from the dataset prior to the analysis because they had missing values?
	6.3. Missing value methods
7. Model development	7.1. Machine leaning models
	7.2. Baseline models
	7.3. Pre-processing
	7.4. Were features selected prior to the actual analysis?
	7.5. Feature selection methods
	7.6. Hyperparameter selection method
	7.7. Ensemble techniques
8. Model performance	8.1. Calibration
	8.2. Discrimination
	8.3. Classification
	8.4. Best performing model
9. Model evaluation	9.1. Internal validation
	9.2. External validation
	9.3. Update
	9.4. Code availability

### Risk of bias assessment

The PROBAST^31^ (Prediction Model Risk of Bias Assessment Tool) was used to independently assess the risk of bias and applicability of selected studies across four domains: participants, predictors, outcomes, and statistical analysis. The full risk of bias assessment results is available in Supplementary material online, files  *S2*.

### Effect measures

The primary outcome of interest in this review is model performance in terms of discrimination. We initially intended to also report on the synthesis of calibration measures; however, the studies utilized a variety of calibration measurements such as calibration slope, calibration plots/curves, Hosmer–Lemeshow test, and Brier score. It is challenging to synthesize a common calibration measurement due to this variety.

Other classification measures such as accuracy, precision, sensitivity, and specificity are not consistently reported by all studies. Another issue is that the calculation of these effect measures requires specifying a diagnostic threshold, which can vary across studies.

Discrimination is most commonly assessed using the c-statistic, also called AUROC (area under the receiver operating characteristic curve), which provides a single measure summarizing the performance of the models across all possible threshold values. This measure reports the trade-off between sensitivity (true positive rate) and specificity (false positive rate) and offers an overall assessment of model performance.

### Data synthesis and statistical analysis

After extracting the required items mentioned previously, we first qualitatively report the characteristics of the selected studies to identify and deduce common settings during the ML-based model development process. Secondly, we report any differences in the methodology settings across model developments to identify possible reasons for heterogeneity across models. Summary statistics will be provided, with continuous items reported as means with standard deviations (SD), and categorical items as percentages.

We extracted the discrimination performance for all ML-based models. Additionally, if a study included baseline conventional models and used the same dataset for training and validation, we also extracted these data. We retrieved the c-statistic/AUROC from the validation/test set, including any standard errors (SEs). If the selected studies failed to report the SE, we used the available confidence interval (CI) to calculate it. If studies reported the same model but with varying settings, we selected the one with the optimal discrimination performance.

To avoid the ‘double counting’ issue mentioned by Hussein H *et al*.^32^ we only retain one model for each ML or conventional approach from data sources that may overlap in the individuals included. The prioritization is based on our judgment of the size of the dataset used, the years of follow-up in the study, and the model's methodological design itself.

For meta-analysis, we used the random effects model^33^ since we anticipated significant heterogeneity across models. We classified ML-based models into different subgroups based on their nature (e.g. ensemble boosting, DL). Additionally, we categorized conventional methods into different subtypes (e.g. QRISK, logistic regression, Cox). We did not consider LR (logistic regression) as an ML-based model in this analysis since most studies use it as a baseline model. Moreover, for well-known conventional risk scores like QRISK, Framingham, SCORE, and ASCVD/PCE, we only identify them when studies are exactly replicating these scores. For example, a self-developed Cox model using Framingham features will be considered a Cox model rather than a Framingham risk score.

For each model subgroup, we report the pooled c-statistic/AUROC, accompanied by its 95%CI. Additionally, we provide the corresponding z-score and *P*-value to indicate the significance of the pooled results. We also generate forest plot figures for each model subgroup to illustrate the results across studies and overall performance (see Supplementary material online, files  *S4*).

Cochran’s Q^34^ with its corresponding *P*-value and the I^2^ statistic^34^ with a 95% CI are reported to assess heterogeneity across each ML models group. Egger’s test^35^ and Begg’s test,^36^ along with their significance levels, are used to assess potential publication bias. Publication bias is also evaluated using funnel plots for each model group (see Supplementary material online, files  *S4*). This meta-analysis was carried out using statistical software called MedCalc.^37^

## Results

### Study selection

**Figure 1:**
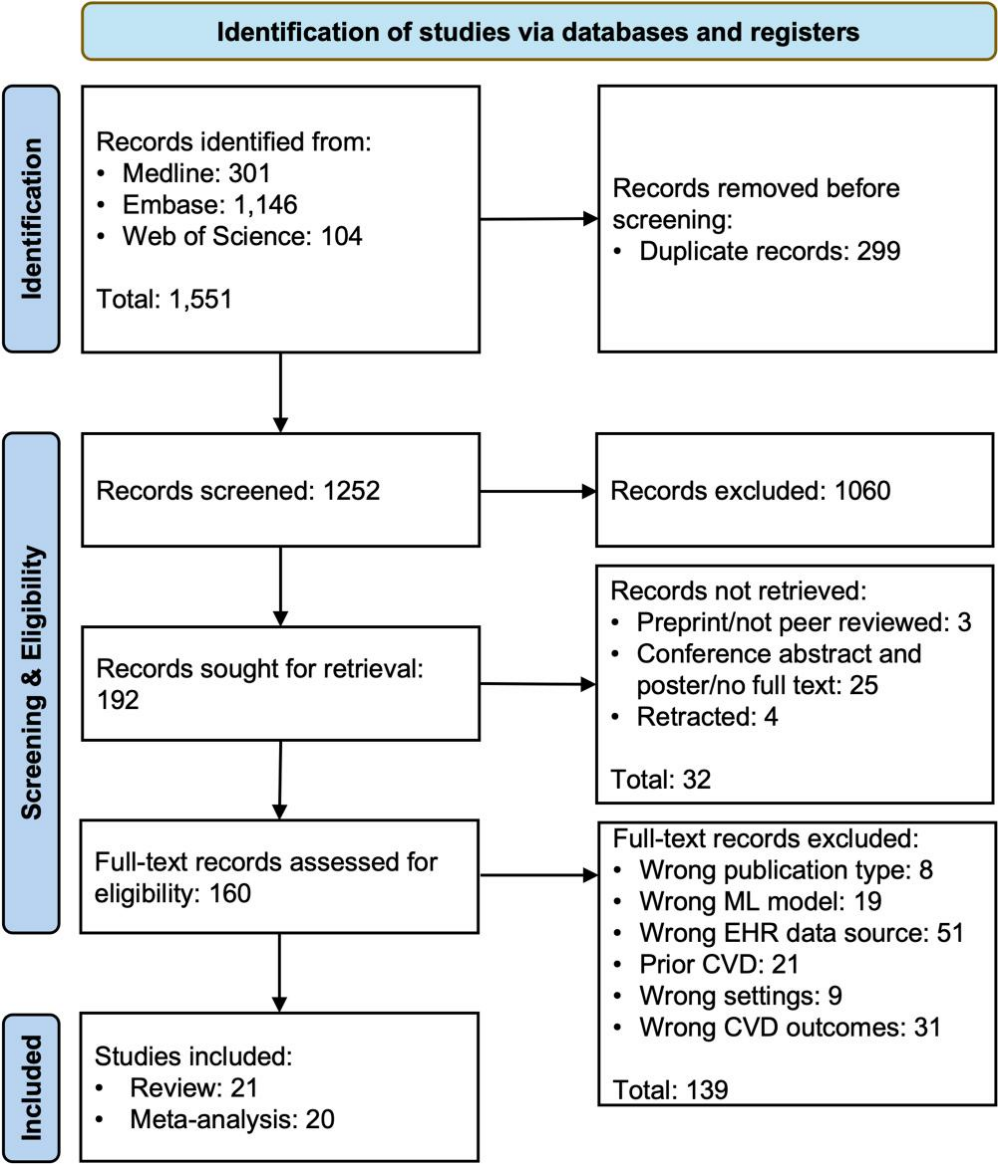
The preferred reporting items for systematic review and meta-analysis flow-chart.

*Figure 1* shows the flow diagram of this study. In total, we identified 1551 publications, reviewed 160 full texts, and finally included 21 studies. Out of 160 full texts reviewed, four studies predicted the CVD outcome with too short a follow-up window^38–41^. Two studies predicted atrial fibrillation^42,43^ (AF), and one study predicted MI,^21^ but use other CVD outcomes as predictors. We also excluded studies that used MIMIC III^44^ (Medical Information Mart for Intensive Care) since it is EHR data but in an intensive care setting. Additionally, we excluded studies that developed models using open data on sharing platforms or repositories such as the heart disease dataset from UCI (University of California, Irvine) ML Repository^45^ and CVD, Framingham, stroke, and HF dataset from Kaggle.^46^ This is because, firstly, they do not have a clear definition of the CVD outcome used and, secondly, they publish baseline model performance and sample code on their website. One study^47^ was not included in the meta-analysis to avoid ‘double counting’ because all its reported models are found in another study^48^ using the same dataset (UK Biobank) with a larger sample size.

### Characteristics of included studies

**Figure 2:**
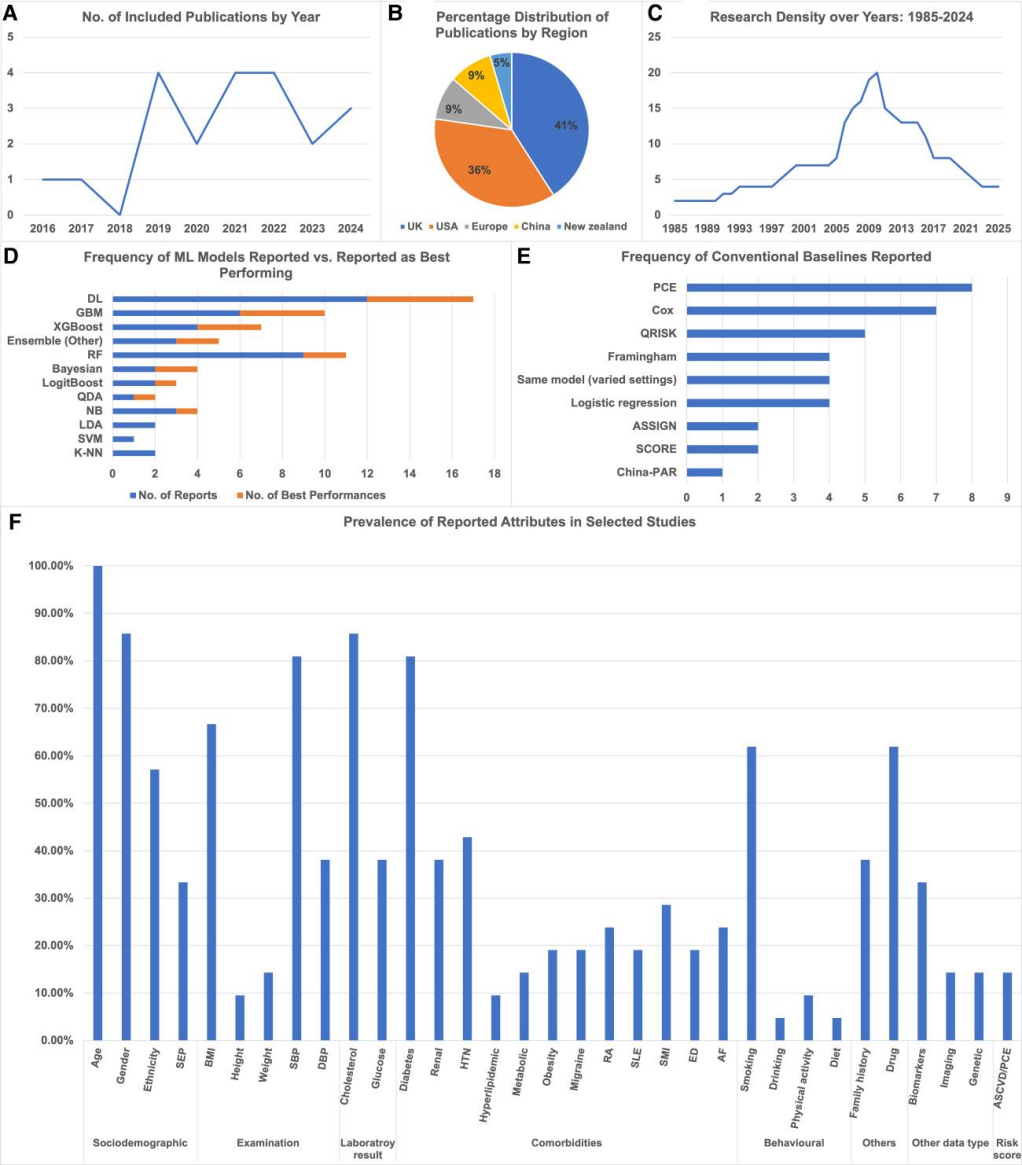
Characteristics of included studies (*A*) annual publications trend; (*B*) regional distribution of publications pie chart; (*C*) research density over the data period: 1985–2024; (*D*) number of selected studies reporting machine learning models and reported as best performing models; (*E*) number of selected studies reporting conventional baselines; (*F*) distribution of features selection in selected publications.

*Table 4* (see Supplementary material online, files  *S1* and *S5*) shows the characteristics of the 21 selected studies. As indicated in *Figure 2A*, these studies spanning from 2016 to 2024. We have observed a rising interest in related papers, particularly throughout the past 5 years.

**Table 4:** Characteristics of included studies

Title (Author year)	Journal	EHR source (Country)	Sample size (cases/patients)	CVD outcomes (follow-up year)	No. of features	ML models (best performing models*)	Baseline models
Machine Learning Adds to Clinical and CAC Assessments in Predicting 10-Year CHD and CVD Deaths^65^	JACC: Cardiovascular Imaging	The CAC Consortium (USA)	66 636 (1.16%)	CHD/IHD/CAD CVD death (10)	77	LogitBoost*	Logistic regression ASCVD/PCE Same model (varied settings)
A novel attention-based cross-modal transfer learning framework for predicting cardiovascular disease^60^	Computers in Biology and Medicine	Vanderbilt University Medical Center (USA) PTB Diagnostic ECG Database Gene Expression Omnibus Database (USA)	109 490 (NR)	All-CVD, not specific (5)	88	Attention-based cross model*	Same model (varied settings)
Machine learning-based marker for coronary artery disease: derivation and validation in two longitudinal cohorts^59^	The Lancet	BioMe (USA) Biobank (UK)	35 749 (14%)	CHD/IHD/CAD (5)	282	Random forest*	ASSIGN
Automating and improving cardiovascular disease prediction using Machine learning and EMR data features from a regional healthcare system^63^	International Journal of Medical Informatics	St. Elizabeth Health Care System (USA)	101 110 (17.39%)	CHD/IHD/CAD Stroke/TIA PAD (10)	28	Neural networks Random forest* Naive Bayes	ASCVD/PCE
Evaluating and mitigating bias in machine learning models for cardiovascular disease prediction^49^	Journal of Medical Informatics	Vanderbilt University Medical Center (USA)	109 490 (9%)	CHD/IHD/CAD Stroke/TIA MI/Heart attack (7)	53	Random forest GBM*	ASCVD/PCE
Machine learning and atherosclerotic cardiovascular disease risk prediction in a multi-ethnic population^63^	Digital Medicine	EHR in Northern California (USA)	131 721 (1.69%)	CHD/IHD/CAD Stroke/TIA MI/Heart attack (5)	10	Random forest GBM* XGB	ASCVD/PCE
Consistency of variety of machine learning and statistical models in predicting clinical risks of individual patients: longitudinal cohort study using cardiovascular disease as exemplar^53^	BMJ	Clinical Practice Research Datalink (UK)	3 661 932 (3.2%)	CHD/IHD/CAD Stroke/TIA (10)	21	Neural networks Random forest GBM*	Qrisk
Predicting cardiovascular risk from national administrative databases using a combined survival analysis and deep learning approach^66^	International Journal of Epidemiology	New Zealand routine national health database (New Zealand)	2 164 872 (W: 2.1%, M: 3.7%)	All-CVD, not specific (5)	20	Neural networks*	Cox proportional hazards
Machine learning to predict cardiovascular risk^62^	International Journal of Clinical Practice	ESCARVAL RISK clinical practice cohort (Spain)	38 527 (NR)	Stroke/TIA CVD death (5)	9	Neural networks K-NN random forest Naive Bayes SVM AdaBoost QDA* LDA	SCORE
Long-Term Exposure to Elevated Systolic Blood Pressure in Predicting Incident Cardiovascular Disease: Evidence from Large-Scale Routine Electronic Health Records^54^	Journal of the American Heart Association	Clinical Practice Research Datalink (UK)	80 964 (3.98%)	CHD/IHD/CAD Stroke/TIA (10)	8	Bayesian analysis*	Cox proportional hazards
A Naive Bayes machine learning approach to risk prediction using censored, time-to-event data^50^	Statistics in Medicine	HMO Research Network Virtual Data Warehouse (USA)	87 363 (4.18%)	Stroke/TIA MI/Heart attack CVD death (5)	5	Naive Bayes*	Framingham
Neural network-based integration of polygenic and clinical information: development and validation of a prediction model for 10-year risk of major adverse cardiac events in the UK Biobank cohort^37^	Lancet Digital Health	Biobank (UK)	395 713 (7.1%)	Stroke/TIA MI/Heart attack CVD death (10)	21	Neural networks*	Cox proportional hazards Same model (varied settings) Qrisk SCORE ASCVD/PCE
Can machine learning improve cardiovascular risk prediction using routine clinical data?^55^	PLOS One	Clinical Practice Research Datalink (UK)	378 256 (6.6%)	All-CVD, not specific (10)	30	Neural networks* random forest GBM	ASCVD/PCE
Cardiovascular disease risk prediction using automated machine learning: A prospective study of 423 604 UK Biobank participants^58^	PLOS One	Biobank (UK)	423 604 (1.58%)	CHD/IHD/CAD Heart failure Stroke/TIA (5)	473	Random forest GBM SVM AdaBoost AutoPrognosis*	Cox proportional hazards Framingham
Actionable absolute risk prediction of atherosclerotic cardiovascular disease based on the UK Biobank^48^	PLOS One	Biobank (UK)	464 547 (6.1%)	CHD/IHD/CAD Heart failure Stroke/TIA MI/Heart attack (10)	203	Random forest XGB*	Logistic regression Qrisk Framingham
Learning from Longitudinal Data in Electronic Health Record and Genetic Data to Improve Cardiovascular Event Prediction^51^	Scientific Reports	Vanderbilt University Medical Center (USA)	109 490 (8.97%)	CHD/IHD/CAD Stroke/TIA (10)	40	CNN RNN random forest GBM*	Logistic regression same model (varied settings) ASCVD/PCE
Machine Learning-Based Risk Prediction for Major Adverse Cardiovascular Events^64^	Studies in Health Technology and Informatics	Steiermärkische Krankenanstaltengesellschaft m.b.H. (Austria)	128 000 (22.86%)	CHD/IHD/CAD Stroke/TIA CVD death (5)	826	Random forest GBM* LDA	NR
Development and Validation of a Bayesian Network-Based Model for Predicting Coronary Heart Disease Risk from Electronic Health Records^52^	Journal of the American Heart Association	Weihai Municipal Hospital (China)	169 692 (6.42%)	CHD/IHD/CAD (5)	11	K-NN weighted survival Bayesian network*	Cox proportional hazards
Validation of risk prediction models applied to longitudinal electronic health record data for the prediction of major cardiovascular events in the presence of data shifts^56^	European Heart Journal	Clinical Practice Research Datalink (UK)	1 003 554 (10.3%)	CHD/IHD/CAD Heart failure Stroke/TIA (5)	21	Deep learning (BEHRT)* Random forest	Cox proportional hazards Qrisk Framingham ASSIGN
Improving cardiovascular risk prediction through machine learning modelling of irregularly repeated electronic health records^87^	European Heart Journal	Chinese Electronic Health Records Research in Yinzhou (China)	215 774 (2.86%)	CHD/IHD/CAD Stroke/TIA MI/Heart attack (5)	25	XGB*	Logistic regression China-PAR
Selection of 51 predictors from 13 782 candidate multimodal features using machine learning improves coronary artery disease prediction^47^	Patterns	Biobank (UK)	13 782 (3%)	CHD/IHD/CAD MI/Heart attack CVD death (10)	51	XGB*	Cox proportional hazards Qrisk ASCVD/PCE

CAC, Coronary calcium scan; CHD/IHD/CAD, coronary heart disease/ischaemic heart disease/coronary artery disease; ASCVD, atherosclerotic cardiovascular diseases; PCE, pooled cohort equation; NR, not reported; TIA, transient ischaemic attack; PAD, peripheral arterial disease; MI, myocardial infarction; GBM, gradient boosting machine; SVM, support vector machine; QDA/LDA, quadratic/linear discriminant analysis; K-NN, *k*-nearest neighbours; XGB, eXtreme gradient boosting; CNN, convolutional neural networks; RNN, recurrent neural networks

The selected studies primarily utilized EHR data from Western countries: the UK (nine studies, 42.86%), the USA (eight studies, 38.10%), and Europe (two studies, 9.52%), with an additional two from China and one from New Zealand (*Figure 2B*).

As for the EHR data sources, four^49–52^ (19.05%) are from single centres, and the rest are from multiple centres. Nineteen (90.48%) studies feature inpatient EHR data, and 17 (80.95%) studies include outpatient data, with 15 (71.43%) studies containing both inpatient and outpatient EHR. It is noteworthy that among the nine UK studies, four use CPRD^53–56^ and four use Biobank^47,48,57,58^ as their training data sources. Another study^59^ use Biobank as external validation data set (see Supplementary material online, files  *S1*).

As shown in *Figure 2C*, the follow-up period for the included studies primarily ranges from 2005 to 2015. The follow-up window is either 5 years or 10 years, with an average of 7.24 (SD 2.49) years. We observe that the sample sizes vary greatly, averaging 470 965 patients (SD 875 138.11), with a range from 13 782 to 3 661 932 patients.

Eighteen (85.71%) studies explicitly excluded patients with prior CVD before the baseline, while the remaining 3^60–62^ (14.29%) studies included patients without prior CVD but did not explicitly report this. Additionally, six^53–55,57,59,63^ (28.57%) studies excluded patients on blood pressure medication, while the others did not specify this criterion (see Supplementary material online, files  *S1*).

All of the selected studies report the development of one or more ML models (*Table 4*). Fifteen (71.43%) of them include comparisons across different ML models, and 17 (80.95) compare these with existing conventional CVD risk scores. Among the ML models, DL has been most frequently reported by the studies, with 12 (57.14%) mentions. Ensembles are also popular, including gradient boosting machine (GBM, six studies, 28.57%), eXtreme Gradient Boosting (XGB, four studies, 19.05%), LogitBoost (two studies, 9.52%), random forest (RF, nine studies, 42.86%), and others (3 studies, 14.29%). Furthermore, DL and ensembles are often reported as the optimal models in the included studies; DL was reported to have the best performance in five (23.81%) studies, while GBM and XGB were reported as best in four (19.05%) and three (14.29%) studies, respectively. *Figure 2D* shows the number of times ML models have been identified as the optimal model with the best performance.

Regarding baseline models (*Figure 2E*), we observe that only one study^64^ did not compare the ML models either with conventional models or with different settings within itself. The most popular existing scores used as baselines are ASCVD/PCE (eight studies, 38.10%), Qrisk (five studies, 23.81%), and Framingham (four studies, 19.05%). The most common conventional models used as baselines are Cox model (seven studies, 33.33%) and LR (four studies, 19.05%).

For the CVD outcomes, studies may use one or more outcomes, which may overlap in definition. Coronary Heart Disease (CHD) was mentioned in 15 studies, stroke/TIA (transient ischaemic attack) 14 times, and MI/heart attack seven times. Most of the studies combined these three CVD outcomes. Nineteen (90.48%) studies had an unbalanced (<30%) CVD outcome in the EHR dataset, with the mean percentage of CVD outcomes per event at 7.48% (SD 0.06).

Eighteen studies (85.71%) reported the complete set of features used, with an average of 109.62 features (SD 199.94), ranging from a minimum of five predictors to a maximum of 826. Studies^48,58,59,64^ that incorporated genetic data and biomarkers tended to report a larger number of features. Excluding these, most studies utilized approximately 30 features. *Figures 2F* show the types of features included in the selected studies. Commonly used sociodemographic features included age (21 studies, 100%), gender (18 studies, 85.71%), and ethnicity (12 studies, 57.14%); examinations included body mass index (BMI, 14 studies, 66.67%), systolic blood pressure (SBP, 17 studies, 80.95%); and laboratory results often featured cholesterol (18 studies, 85.71%). Among the comorbidities, diabetes (17 studies, 80.95%), hypertension (nine studies, 42.86%), and kidney disease (eight studies, 38.10%) were the top three utilized in the models. Smoking (13 studies, 61.90%) was the most frequently reported behavioural risk factor. Additionally, family history of CVD (eight studies, 38.10%) and drugs (13 studies, 61.90%), including hypertensive drugs, were also commonly used. We also identified some models that incorporated other data types, including biomarkers (seven studies, 33.33%), imaging (three studies, 14.29%), and genetic features (three studies, 14.29%). Three studies^61,63,65^ (14.29%) directly used the ASCVD/PCE risk score as one of their features. Seventeen studies (80.95%) report on how they handled missing values. Although many ML models can use data with missingness, 11 studies (52.38%) still reported using single (mean, median) or multiple (chained equation) imputation methods (see Supplementary material online, files  *S1*).

For model development, 13 studies (61.90%) manually selected features based on expertise or previous research, while the remaining eight (38.10%) used data-driven techniques including χ^2^, Lasso, and RF for feature selection. Nine studies (42.86%) employed grid search and four used random search for hyperparameter selection. One study^66^ (4.76%) utilized Bayesian optimization, but seven studies (33.33%) did not mention this procedure. Many researchers used ensemble methods; we observed that 14 studies (66.67%) implemented bagging, nine (42.86%) used boosting, and one study^62^ (4.76%) applied stacking (see Supplementary material online, files  *S1*).

Regarding model performance reporting, seven studies (33.33%) did not report any calibration results. Six (28.57%) used calibration plots/curves, four provided a calibration slope, three (14.29%) conducted the Hosmer–Lemeshow test, and six studies (28.57%) reported the Brier score. All studies reported discrimination performance, using Harrell’s C-statistic/AUROC. For classification, the most frequently used performance metrics are specificity (eight studies, 38.10%), sensitivity (nine studies, 42.86%), and precision (seven studies, 33.33%). Four studies (19.05%) comparing ML with conventional methods also reported the net reclassification improvement (NRI). Since classification measures require a cut-off threshold, only eight studies reported their cut-off threshold selection (see Supplementary material online, files  *S1*).

For validation, nearly all the studies used *n*-fold cross-validation and split the dataset into training and testing phases, with some adding a validation set. We found three studies^49,56,59^ (14.29%) that used external validation spatially, among which one^56^ (4.76%) also conducted temporal external validation.

Ten studies (47.62%) have shared their code on public platforms like GitHub, but only three studies^56,57,64^ (14.29%) claimed they followed guidelines for model development, typically those outlined in TRIPOD.^67^

### Risk of bias in studies

**Figure 3:**
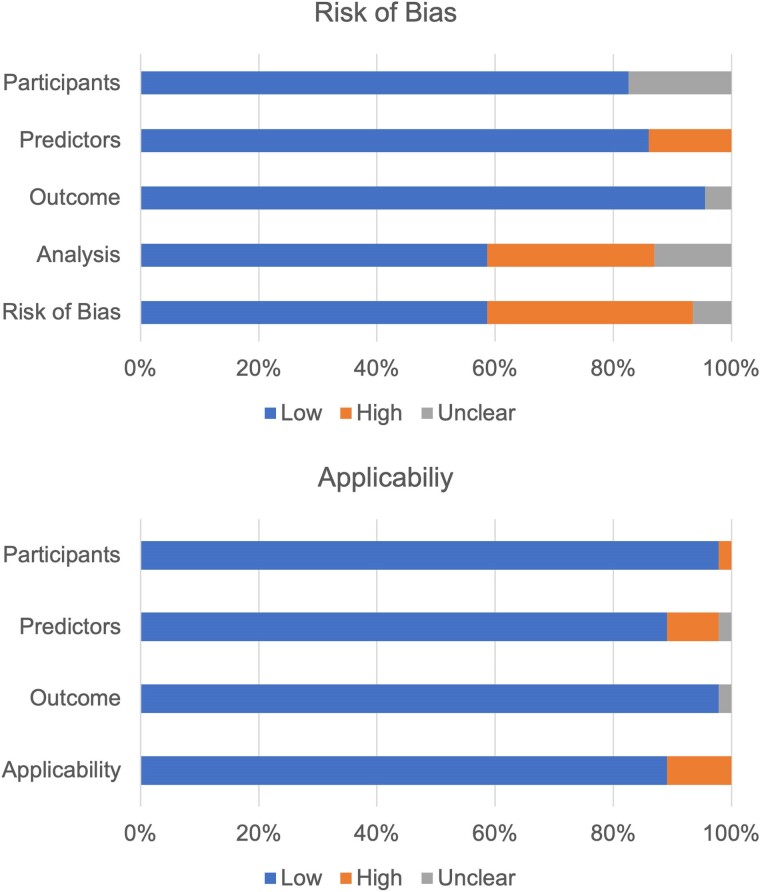
Summary of ROB and applicability assessment using PROBAST.

As shown in *Figure 3*, we identified 16 (34.5%) ML models with a high risk of bias, mainly due to issues in the analysis domain (13 models, 28.3%), particularly with predictors pre-processing, handling missing data, complexities in the data, and model overfitting. The reporting in the analysis domain was often inadequate, either missing or lacking sufficient methodological information. Another six (13.0%) ML models exhibited a high risk of bias in the predictor’s domain.

Applicability was generally acceptable (41 models, 89.1%) across the ML models, as we were relatively strict in study selection. The participants were from a valid EHR source, and the predictors were generally based on previous research or expertise, with a clear definition of CVD outcomes using ICD codes. For those that did not provide an exact code for their CVD outcomes, we labelled them as unclear (one models,^59^ 2.2%). Four models^59,64^ (8.7%) are rated high for applicability due to the predictors domain, as they used too many biomarkers, which were not practical enough for the usefulness of the type of model they are designed for.

### Meta-analysis

In total, we retrieved 45 ML models along with 35 baseline conventional models from selected studies, as shown in *Table 5* (see Supplementary material online, files  *S3*). After removing duplicates of models derived from CPRD, UK Biobank, and Vanderbilt University Medical Centre to avoid the ‘double counting’ issue, we retained 32 ML models and 26 baseline conventional models.

**Table 5:** Pooled model performance, heterogeneity, and publication bias results

Model (*n*)	Pooled AUC (95% CI, *P*-value)	Q (*P*-value)	I^2^ (95% CI)	Egger’s test intercept (*P*-value)	Begg’s test Kendall’s Tau (*P*-value)
NB (3)	0.772 (0.721–0.824, *P* < 0.001)	101.14 (*P* < 0.0001)	98.02% (96.34–98.93%)	−8.67 (*P* = 0.5925)	−0.3333 (*P* = 0.6015)
DL (6)	0.847 (0.766–0.927, *P* < 0.001)	7427.98 (*P* < 0.0001)	99.93% (99.92–99.94%)	20.26 (*P* = 0.3984)	−0.0667 (*P* = 0.8510)
RF (6)	0.865 (0.812–0.917, *P* < 0.001)	1674.62 (*P* < 0.0001)	99.70% (99.63–99.76%)	0.7493 (*P* = 0.9422)	−0.2000 (*P* = 0.5730)
XGBoost (3)	0.776 (0.758–0.794, *P* < 0.001)	29.52 (*P* < 0.0001)	93.22% (83.56–97.21%)	4.38 (*P* = 0.1758)	0.3333 (*P* = 0.6015)
GBM (5)	0.821 (0.773–0.869, *P* < 0.001)	954.91 (*P* < 0.0001)	99.58% (99.46–99.68%)	−12.72 (*P* = 0.1128)	−0.1054 (*P* = 0.7963)
Boosting (11)	0.796 (0.763–0.829, *P* < 0.001)	12 864.28 (*P* < 0.0001)	99.92% (99.91–99.93%)	−22.03 (*P* = 0.0679)	0.1101 (*P* = 0.6374)
Ensemble (19)	0.820 (0.790–0.850, *P* < 0.001)	15 356.98 (*P* < 0.0001)	99.88% (99.87–99.89%)	−15.16 (*P* = 0.0496)	−0.1018 (*P* = 0.5424)
ML (32)	0.815 (0.787–0.842, *P* < 0.001)	34 320.25 (*P* < 0.0001)	99.91% (99.90–99.91%)	−11.81 (*P* = 0.0783)	−0.1263 (*P* = 0.3098)
LR (7)	0.796 (0.750–0.842, *P* < 0.001)	12 121.90 (*P* < 0.0001)	99.95% (99.95–99.96%)	−25.85 (*P* = 0.2220)	−0.1429 (*P* = 0.6523)
Cox (6)	0.787 (0.740–0.834, *P* < 0.001)	13 209.49 (*P* < 0.0001)	99.96% (99.96–99.97%)	−40.02 (*P* = 0.1407)	0.1380 (*P* = 0.6973)
PCE (5)	0.760 (0.721–0.798, *P* < 0.001)	877.47 (*P* < 0.0001)	99.54% (99.40–99.65%)	2.83 (*P* = 0.8294)	0.0000 (*P* = 1.0000)
Qrisk (3)	0.780 (0.699–0.860, *P* < 0.001)	15 974.24 (*P* < 0.0001)	99.99% (99.99–99.99%)	−71.81 (*P* = 0.5031)	−0.3333 (*P* = 0.6015)
Conventional (19)	0.765 (0.734–0.796, *P* < 0.001)	57 757.61 (*P* < 0.0001)	99.97% (99.97–99.97%)	−38.32 (*P* = 0.0096)	0.1075 (*P* = 0.5202)

AUC, area under curve; NB, naive Bayes; DL, deep learning; RF, random forest; GBM, gradient boosting machine; SVM, support vector machine; QDA/LDA, quadratic/linear discriminant analysis; K-NN, k-nearest neighbours; XGBoost, eXtreme gradient boosting; CNN, convolutional neural networks; RNN, recurrent neural networks; Cox, cox proportional hazards; LR, logistic regression; PCE, pooled cohort equation.

We only applied the random effects model for those models that were reported more than three times. For ML models, we have DL (*n* = 6) subgroup, which includes NN and self-defined DL models. Other model subgroups include Naive Bayes (*n* = 3), RF (*n* = 6), XGB (*n* = 3), GBM (*n* = 5). The boosting (*n* = 11) subgroup that involves XGB, GBM, two LogitBoost models, and one AdaBoost models. Additionally, there was an ensemble (*n* = 19) subgroup that includes the boosting group, RF, and two other ensemble models. There was also an ML (*n* = 32) group that contains all the aforementioned ML models along with one support vector machine (SVM), one linear discriminant analysis (LDA), one quadratic discriminant analysis (QDA), and one Bayesian models.

For the baseline models, we had LR (*n* = 7), Cox (*n* = 6), PCE (*n* = 5), and QRISK (*n* = 3) subgroup. Additionally, the conventional (*n* = 19) subgroup included one China-PAR, two Framingham, and two SCORE models. The LR model was excluded from the conventional group as it is not comparable to the other conventional risk scores. We did not include ASSIGN since the two studies that used ASSIGN as a baseline reported diverging outcomes: one reported an extremely low AUC,^56^ and the other^59^ did not report the AUC for ASSIGN.

**Figure 4:**
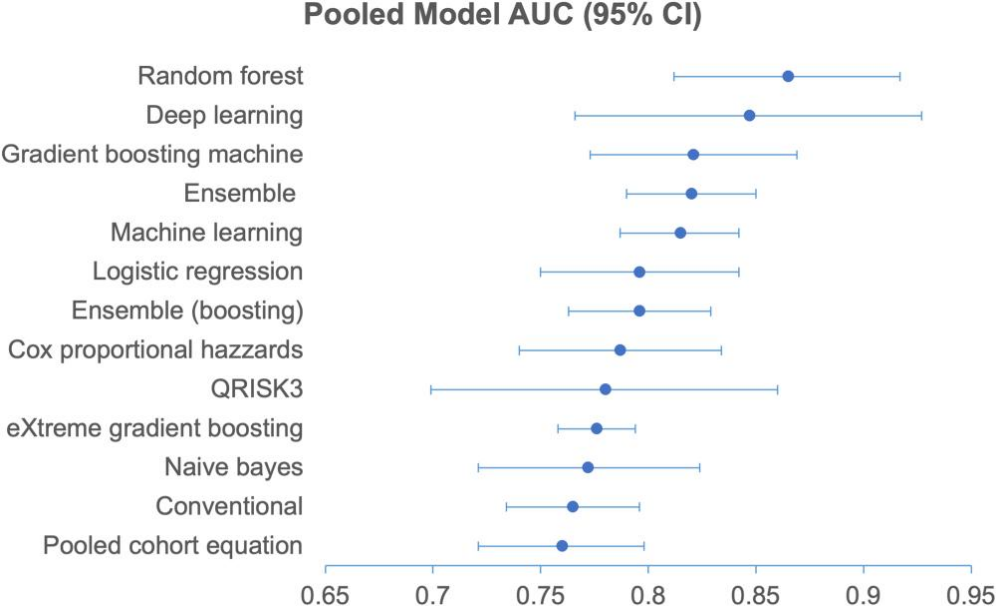
Pooled model AUC with 95% confidence intervals (random effects).

As shown in *Table 5* and *Figure 4*, all the models’ pooled AUCs are statistically significant at *P* < 0.001. Among all models compared, the RF, an ensemble bagging model, performed the best with a pooled AUC of 0.865 (95% CI: 0.812–0.917), followed by DL with 0.847 (95% CI: 0.766–0.927). GBM is the best-performing ensemble boosting model with an AUC of 0.821 (95% CI: 0.773–0.869).

LR performed considerably well among all baseline subgroups with an AUC of 0.796 (95% CI: 0.750–0.842). Cox (0.787, 95% CI: 0.740–0.834) and Qrisk (0.780, 95% CI: 0.699–0.860) also performed relatively well compared with other conventional risk scores. However, the PCE (0.760, 95% CI: 0.721–0.798) was less effective, having the lowest performance among all models.

We observe that ML models generally (0.815, 95% CI: 0.787–0.842), especially ensemble methods (0.820, 95% CI: 0.790–0.850), perform better than conventional baselines (0.765, 95% CI: 0.734–0.796).

Notably, we observed very high heterogeneity across all model subgroups, as indicated by the significantly high *Q* scores. The I² scores confirm this finding; all models have I² scores around 99%, which clearly show significant differences across studies.

The forest and funnel plots for all model subgroups are available in Supplementary material online, files  *S4*.

For the publication bias comparison, the ensemble and conventional model subgroups showed significant publication bias risks (*P*-values of 0.0496 and 0.0096, respectively), as indicated by Egger’s test. Other models had higher *P*-values (>0.05) in Egger’s test, suggesting no significant publication bias. Most models did not show significant publication bias based on Begg’s test, given the high *P*-values. However, the XGB, NB, and QRISK models had relatively higher Kendall’s Tau values (±0.3333), although their *P*-values did not reach statistical significance (>0.05). The Ensemble and conventional models exhibited potential publication bias as indicated by significant results from Egger’s test. Although most models did not show publication bias according to Begg’s test, the results from Egger’s test advise caution.

### Discussion

Our findings are confirmed by several systematic reviews reporting that ML outperforms conventional methods in CVD risk prediction^19,68–71^. Among the ML models, ensembles including RF and boosting are preferreds.^68,69,72,73^ Furthermore, several studies report that DL also performs better than other ML models.^68,69^ However, the CVD tasks they focus on often extend beyond primary prevention of CVD to broader AI applications in CVD risk assessment.^14^ Additionally, the CVD outcomes, settings, and populations vary across the selected models.^69,70,72,73^

While a recent systematic review^19^ reported on AI risk prediction models for CVD, it did not perform a data synthesis across different ML models. Another recent systematic review^24^ shares our interest but extends beyond EHR sources to include structured records from cohorts.

To the best of our knowledge, our review is the first to exclusively discuss ML-based algorithms using EHR for medium to long-term CVD risk prediction for primary prevention. We included only models that had been tested in the general population using EHR, ensuring the applicability of our results for primary prevention in a primary care setting.

Heterogeneity among studies has also been frequently reported by previous systematic reviews.^69,73^ For this issue, we had anticipated challenges but have yet to identify viable solutions. Currently, the only approach we can consider under these circumstances is to recommend that future model development studies report their methodologies with utmost detail. We found that fewer studies utilize guidelines like TRIPOD,^67^ and the same research group has just launched a TRIPOD specification specifically for AI models^29^ . However, we observe a phenomenon where it is evident that researchers employ certain settings and methods, such as excluding patients with prior CVD and some pre-processing procedures, inferred from experience and implicit clues within their studies, yet these are not explicitly reported.

We observe that several studies incorporate genetic and biomarker data as predictor features in their models, often utilizing combined EHR sources with cohorts that include such information, such as the UK Biobank. The use of genetic and biomarker data has been proven to be a promising approach for personalizing predictions, particularly concerning CVD epigenomic information.^74^ However, the algorithms derived from these studies are typically designed for screening and prevention in primary care settings, or even for patients to self-evaluate. In these settings, genetic and biomarker data may be difficult to obtain.

Several models listed incorporate features related to social determinants of health (SDOH), such as deprivation data, socio-economic position (SEP), or environmental factors like air pollution. These features are readily available through self-reporting or can be derived from location data, making them easier to access in practice. This approach can improve the performance of individual predictions within certain population groups and help mitigate health inequalities.^75^

The heterogeneity appears inevitable given the nature of ML models and the complex data structures of EHR. However, it is essential to ensure that the model development process is both transparent and replicable. Data platforms like UCI ML Repository and Kaggle help solve this problem by providing a fair comparison across models via external validation. Researchers can also freely share their code and settings on these platforms. However, models trained on these datasets may exhibit limited clinical applicability since they do not have clear definitions for comorbidities and CVD outcomes. Nevertheless, this approach provides a methodical way to systematically evaluate the features of proposed models. The diversity in outcome code lists used for defining comorbidities and CVD conditions in further study are obstacles to the replicability of these studies. The use of standardised phenotype definitions (e.g. HDR UK National Phenotype Library^76^ or The OHDSI Phenotype Library^77^) can help alleviate the problem to an extent.^78^

We also observe that most of the EHR sources are developed in countries and regions where the white ethnicity predominates. Even when considering some studies that met the inclusion criteria but were not reported, the majority still originated from EHR data in the UK, USA, and Europe, which is also common for traditional cohort studies.^70,72,79,80^ But considering the cost and effort of actively collecting cohort data and follow-up, EHR might be a future option with the development of technology, though access to this source still remains limited in less developed areas.^81^ Alternatively, it may be possible to adapt models originally developed in predominantly white populations for use with minority ethnic groups, potentially enhancing model performance in underdeveloped areas without necessitating extensive EHR management efforts.

During our search, we identified several relevant studies^82–85^ that utilize datasets derived from insurance claims, such as the National Health Insurance Service-Health Screening^86^ (NHIS-HEALS) in South Korea. These datasets have a similar structure to EHRs, representing the secondary use of routinely collected data, and offer comprehensive medical records with broad population coverage and long-term follow-up. However, they are very sensitive to bias stemming from the insured population, national health systems and local claims processes, e.g. NHIS-HEALS only insures people aged 40–79 years. Future studies may also consider integrating this available data source or conducting external validation.

For the studies we included, we haven’t found any clinically applicable study or cost-effectiveness study for them, nor, to our knowledge, do we think they are currently being used in clinical settings at all. More action should be taken to move from development and validation to application.

### Limitation

Our analysis has several limitations. Firstly, despite our rigorous approach to study selection, significant heterogeneity in CVD outcomes remained across the selected studies. Although most studies provided detailed ICD codes to define their CVD outcomes, we observed variations in the terms used to describe the same CVD condition, with several terms overlapping in their definitions. Therefore, the actual codes used may vary even for nominally identical CVD outcomes, especially since some studies broaden their definition to include not only disease incidence but also related procedures and treatments.

Another limitation of our analysis is the inability to report calibration data for the selected studies. As previously discussed, the variation in methodologies used, and in some instances, the complete absence of calibration data, preclude such reporting. Additionally, the limited number of selected studies that fulfil both ML-based criteria and use of EHR for primary prevention of CVD in the long term is due to strict inclusion criteria. Although more studies have been identified in recent years, the numbers are still limited. Moreover, for some self-derived DL models, we have had to categorize them roughly as a DL subgroup, even though they may have different structures in layers and numbers of neurons, due to the limited selection available.

Another limitation arises from the heterogeneity in methodology and data sources, combined with varying sets of features, hyperparameters, and software packages used, making it difficult to authoritatively say that one ML model is better than another. The results of this review can only suggest that some types of models might be preferable over others, and that some models may not be worth pursuing at all. Similarly, in feature selection, some features have been consistently reported to have more predictive power than others. Thus, the only clear conclusion is that further study of these models and features is needed. However, the diversity in hyperparameters settings and the need for further approval from EHR data providers make it very difficult to replicate their work.

### Conclusions

This systematic review and meta-analysis evaluated ML models alongside baseline conventional models utilizing EHR for risk prediction in context of primary prevention of CVD. It was observed that ML models, which increasingly consider factors beyond traditional risk factors such as age, gender, and diabetes but also data sources including image, biomarker, and genetic data outperform conventional risk scores in CVD risk prediction for primary prevention. Particularly, DL and ensemble methods, especially boosting and bagging, may be considered optimal models by researchers.

However, challenges such as a high risk of bias and heterogeneity, the complexity of EHR systems, a lack of external validation and calibration performance reports, and an absence of clinical impact studies raise concerns about the clinical applicability of these ML models for actual use in real-world settings, when compared to established scores such as QRISK and PCE.

Despite their superiority in discrimination, the real-world effectiveness of ML models remains questionable, underscoring the need for more transparent methodology reporting to support validation. The results indicate that the dynamic approaches of ML could pave the way for future developments in predicting CVD risk, but these need to be properly evaluated in clinical settings.

Thus, future efforts should focus on enhancing methodological transparency and replicability and establishing real-world implementation and evaluation techniques.

## Supplementary Material

ztae080_Supplementary_Data

## Data Availability

The data underlying this article are available within the article itself and in the accompanying online supplementary material.

## Appendix

Search strategy

1. cardiovascular disease$.mp. or exp Cardiovascular Diseases/
2. exp Machine Learning/
3. (artificial intelligence or AI or machine learning or deep learning or neural network$).ti,ab.
4. exp Natural Language Processing/
5. (2 or 3) not 4
6. exp Risk Assessment/ or exp Risk Factors/
7. (predict$ or risk or score$ or model$ or algorithm$).ti.
8. 6 or 7
9. exp Electronic Health Records/
10. (electronic health record$ or electronic medical record$).mp.
11. 9 or 10
12. 1 and 5 and 8 and 11
