# Supplementary material for: Machine learning based prediction models for cardiovascular disease risk using electronic health records data: systematic review and meta-analysis
Source: Eur Heart J Digit Health. 2024 Oct 27;6(1):7–22. doi: 10.1093/ehjdh/ztae080 (PMC11750195; doi:10.1093/ehjdh/ztae080)

Naïve bayes

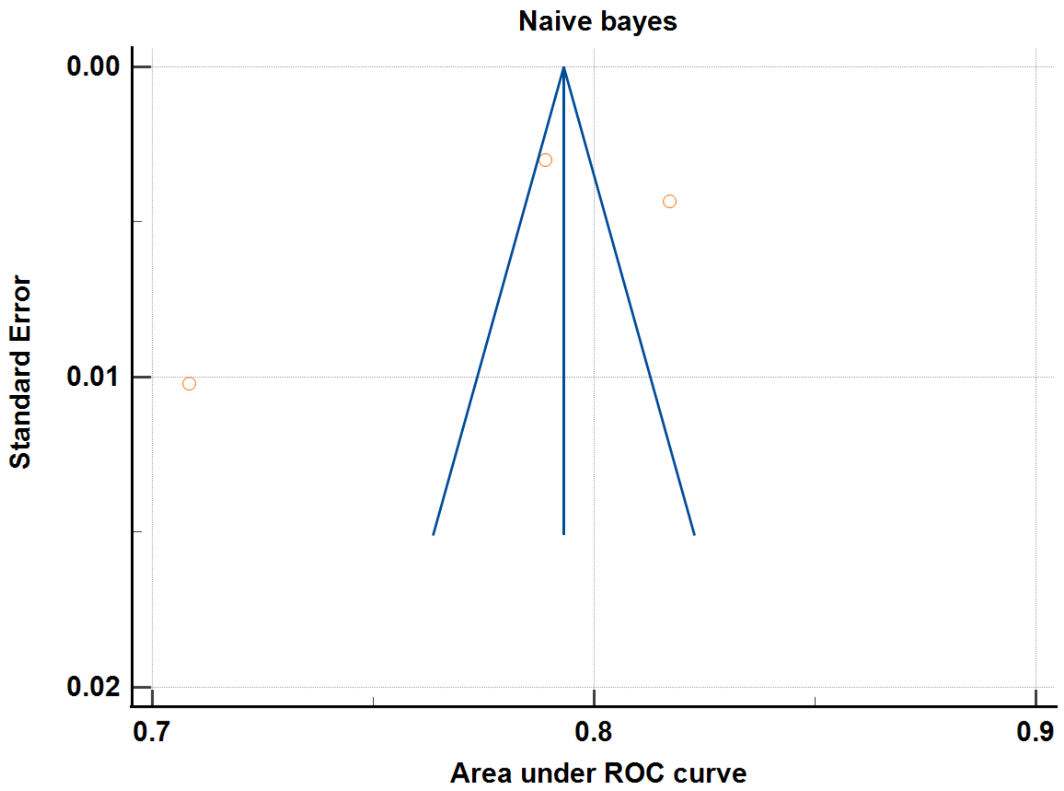
Naive bayes
0.00
0.01
0.02
Standard Error
0.7
0.8
0.9
Area under ROC curve

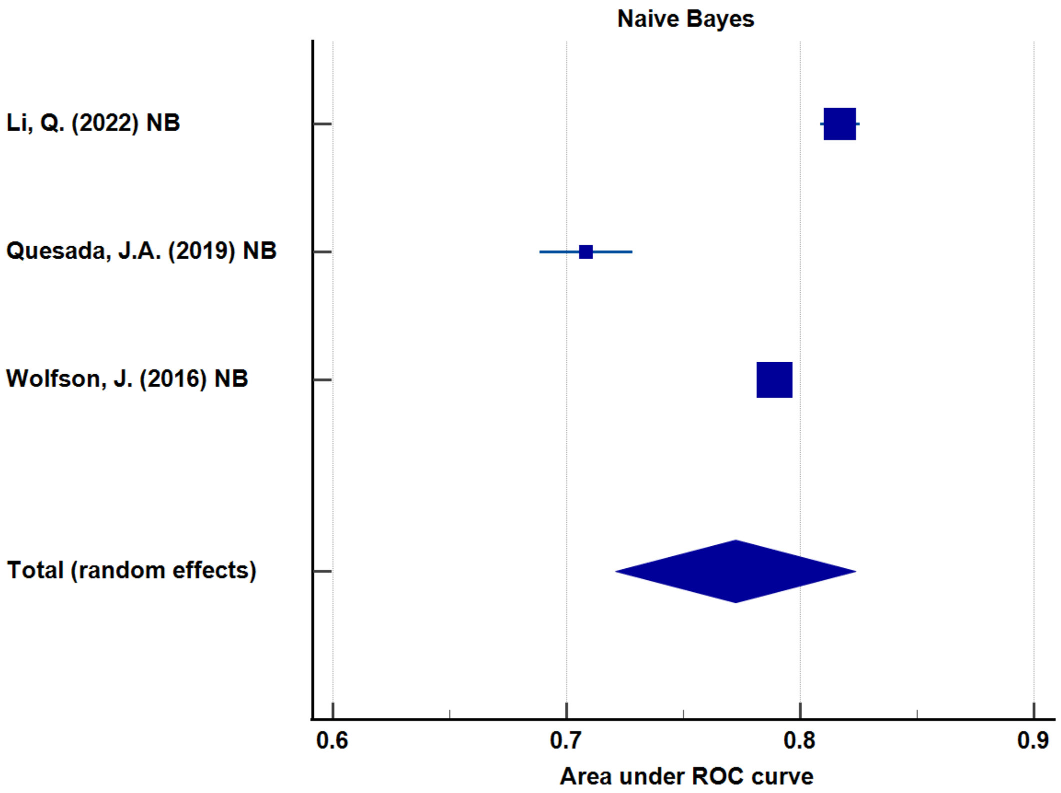
Naive Bayes
Li, Q. (2022) NB
Quesada, J.A. (2019) NB
Wolfson, J. (2016) NB
Total (random effects)
0.6
0.7
0.8
0.9
Area under ROC curve

Deep learning

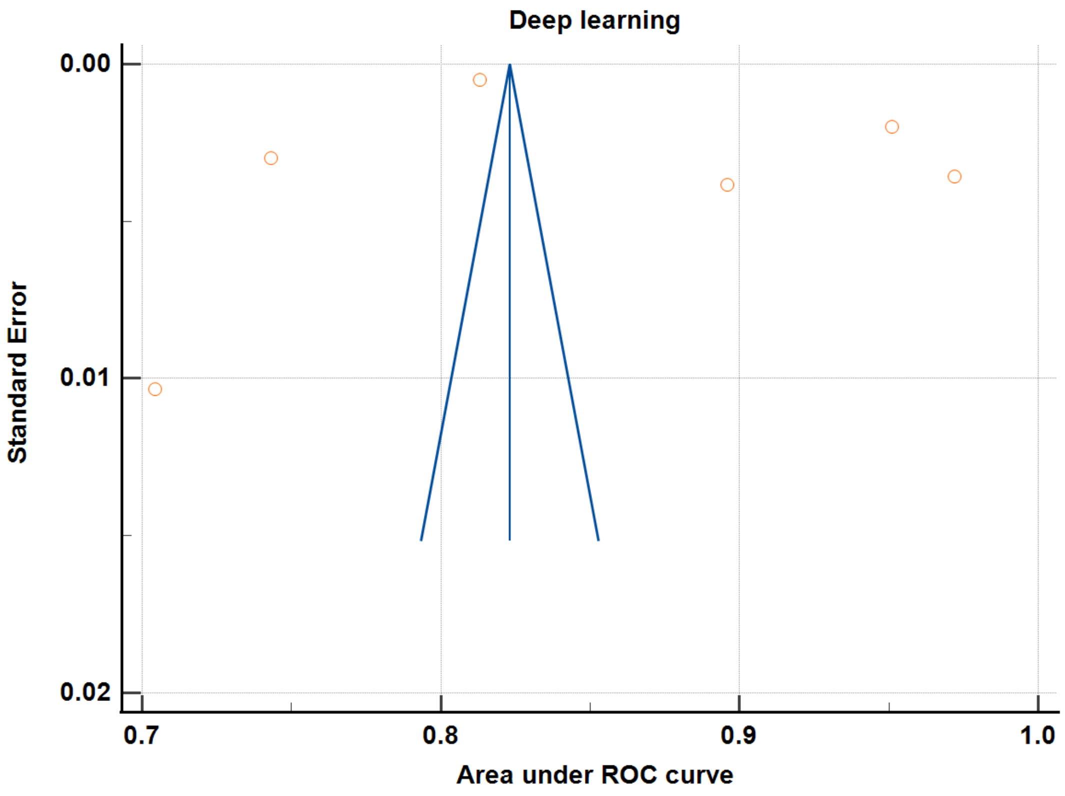

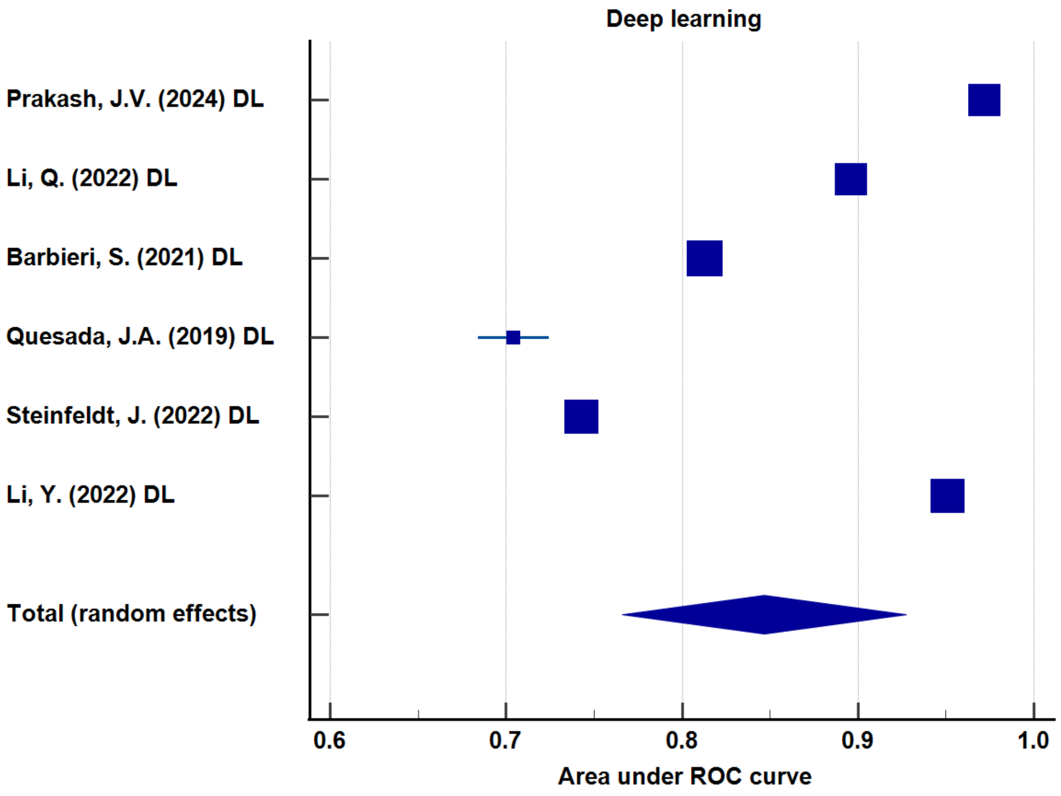

Random forest

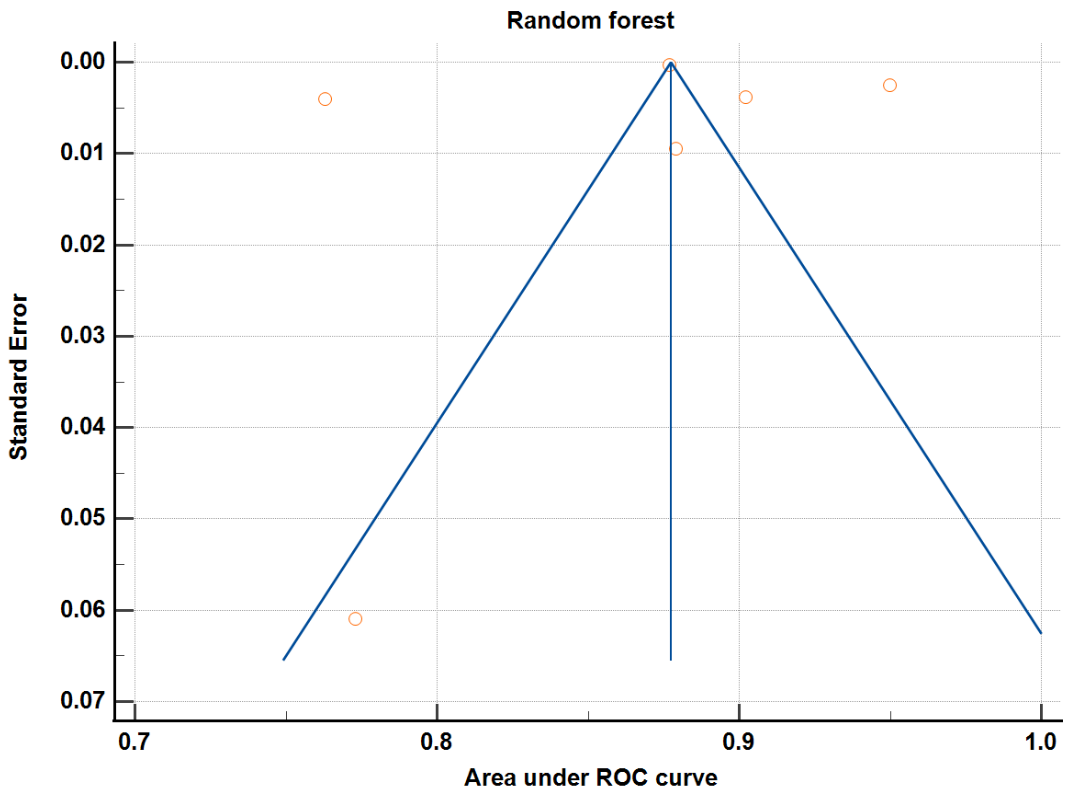

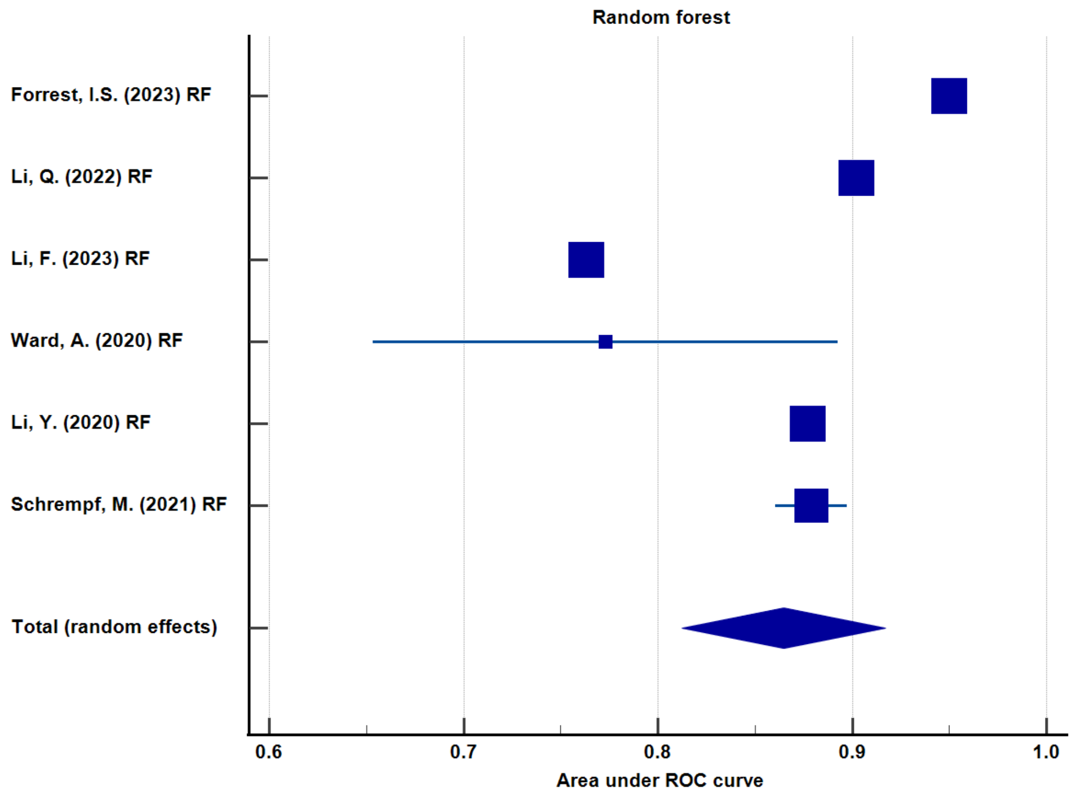

Gradient boosting machine

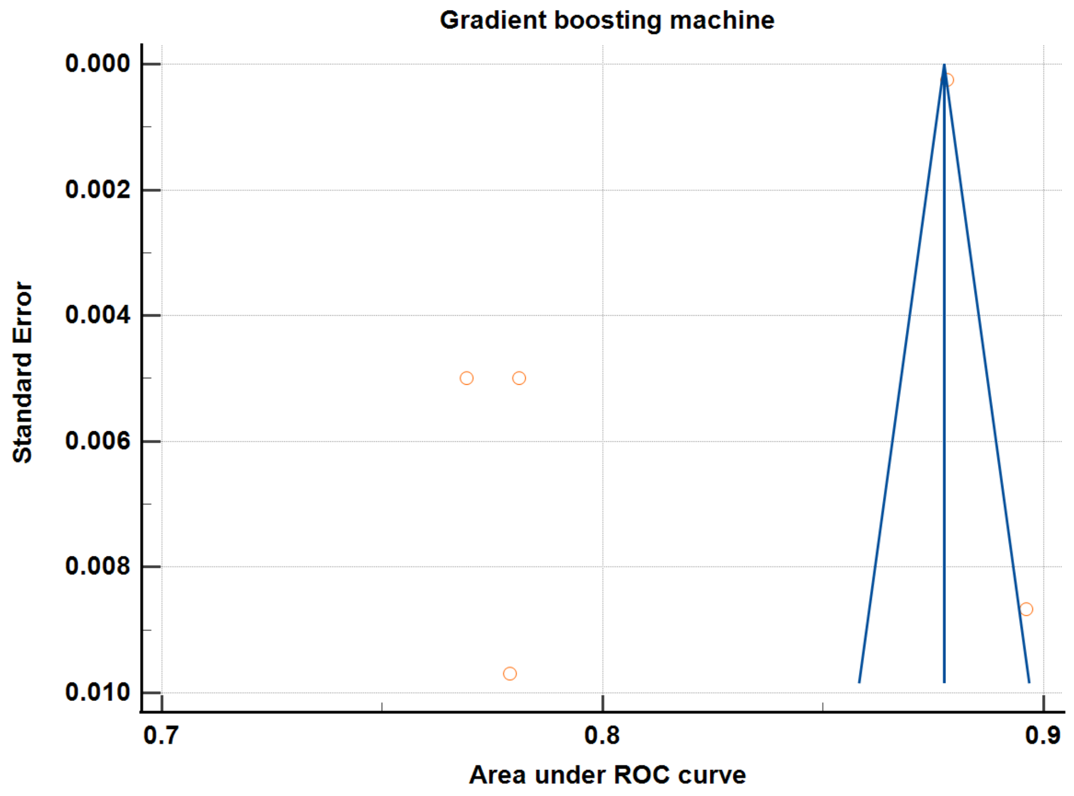

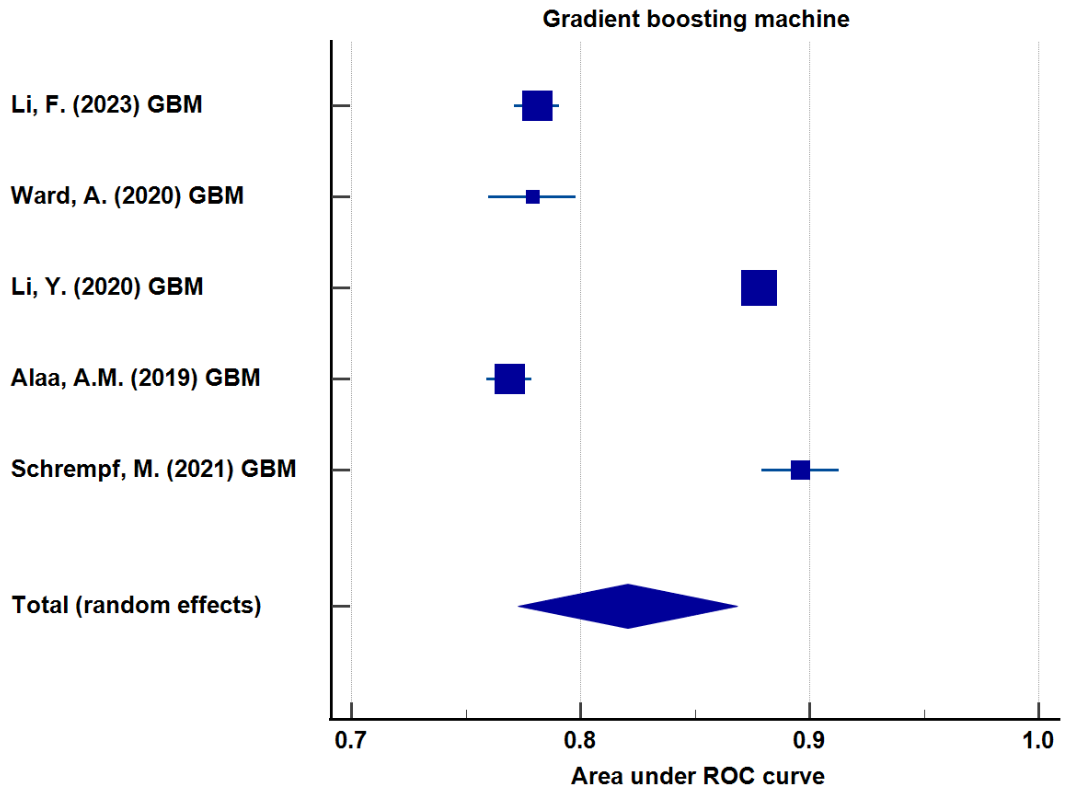

eXtreme boosting machine

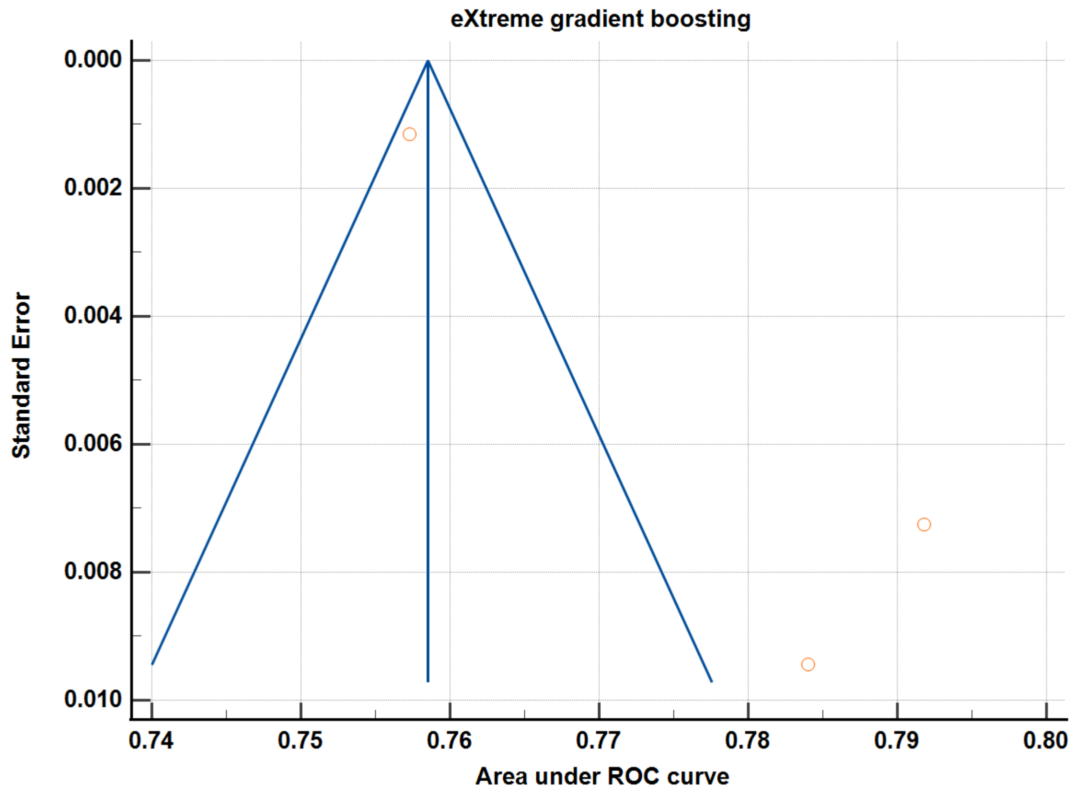

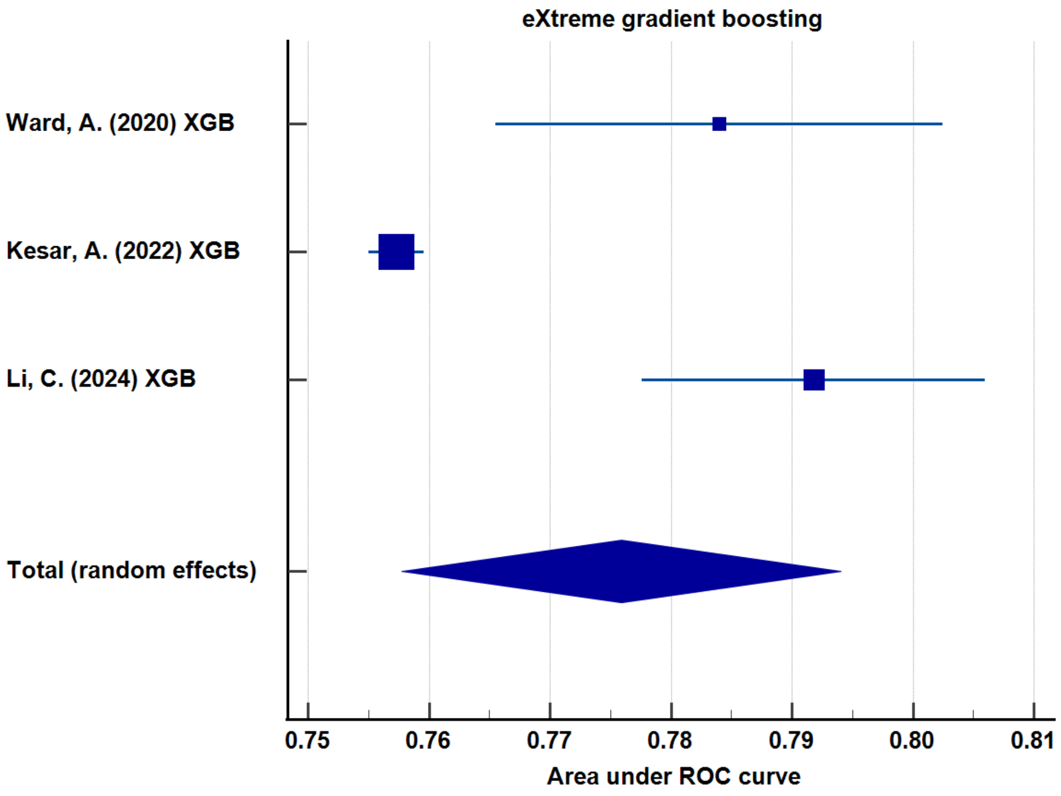

Ensemble

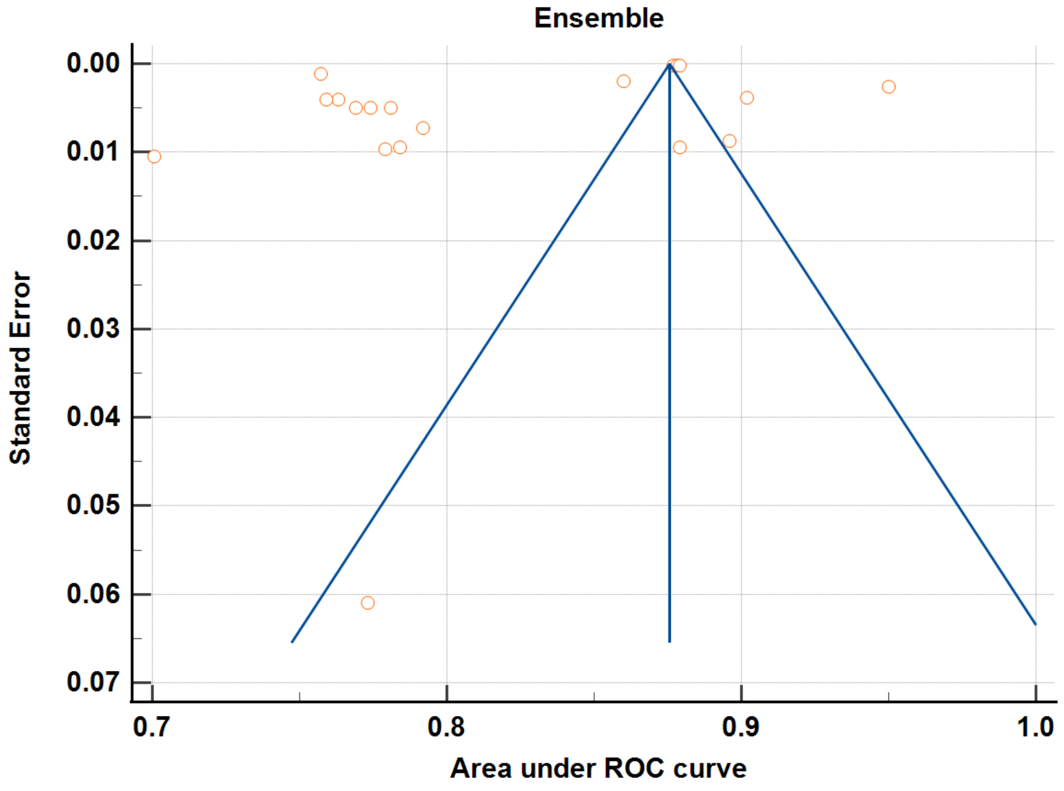

Ensemble
0.00
0.01
0.02
0.03
0.04
0.05
0.06
0.07
Standard Error
0.7
0.8
0.9
1.0
Area under ROC curve

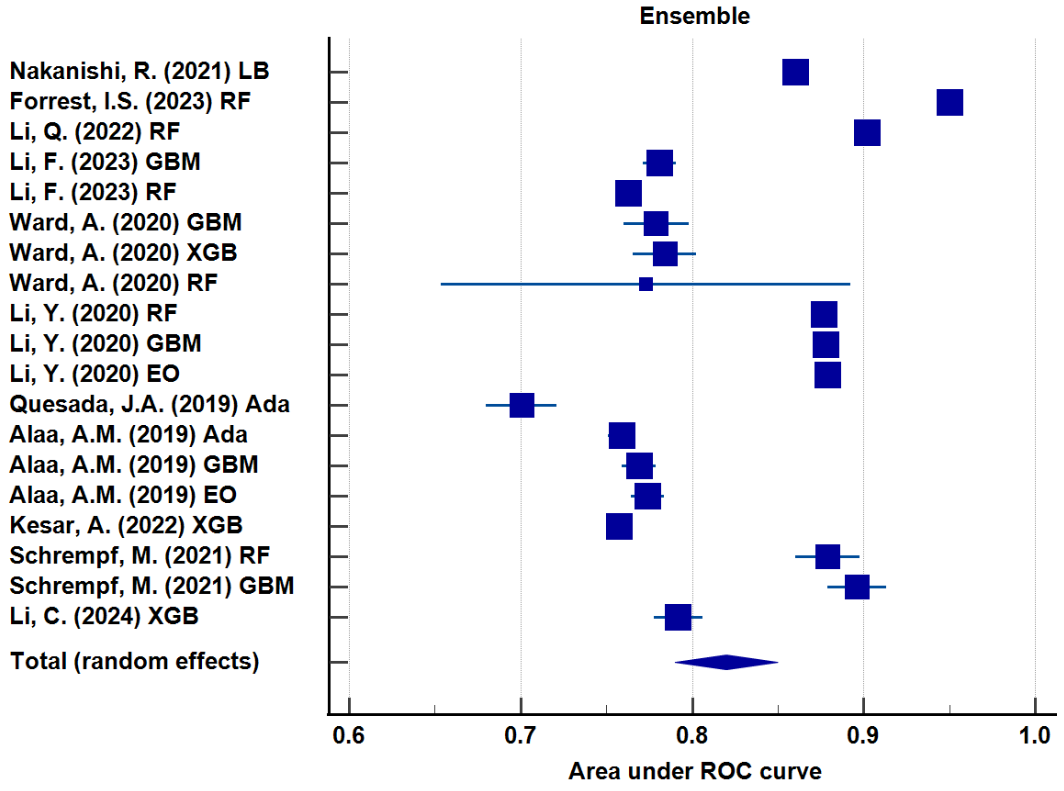

Ensemble
Nakanishi, R. (2021) LB
Forrest, I.S. (2023) RF
Li, Q. (2022) RF
Li, F. (2023) GBM
Li, F. (2023) RF
Ward, A. (2020) GBM
Ward, A. (2020) XGB
Ward, A. (2020) RF
Li, Y. (2020) RF
Li, Y. (2020) GBM
Li, Y. (2020) EO
Quesada, J.A. (2019) Ada
Alaa, A.M. (2019) Ada
Alaa, A.M. (2019) GBM
Alaa, A.M. (2019) EO
Kesar, A. (2022) XGB
Schrempf, M. (2021) RF
Schrempf, M. (2021) GBM
Li, C. (2024) XGB
Total (random effects)
0.6
0.7
0.8
0.9
1.0
Area under ROC curve

Ensemble (boosting)

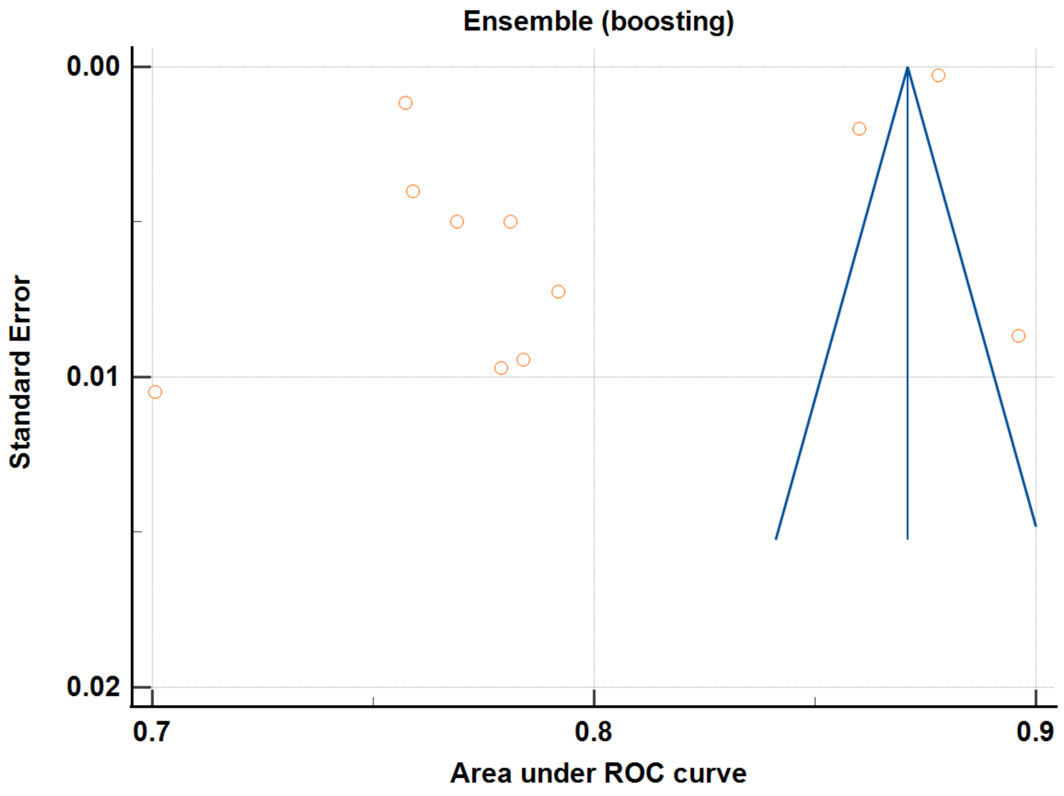

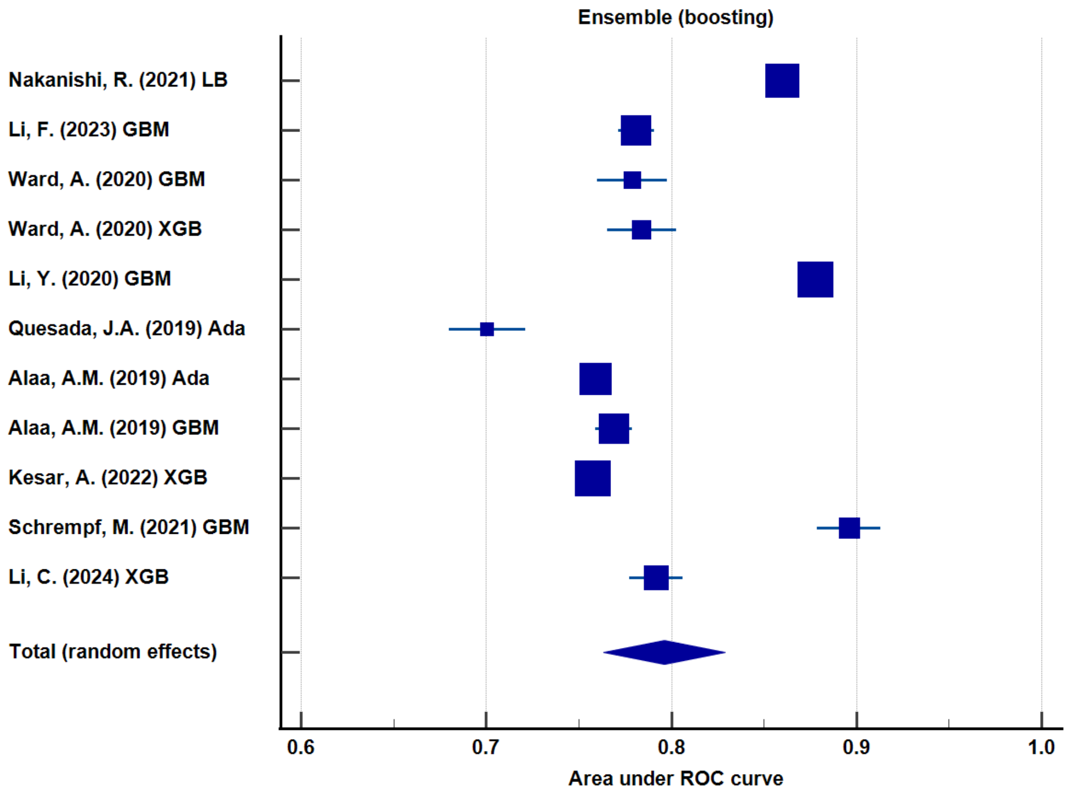

Machine learning

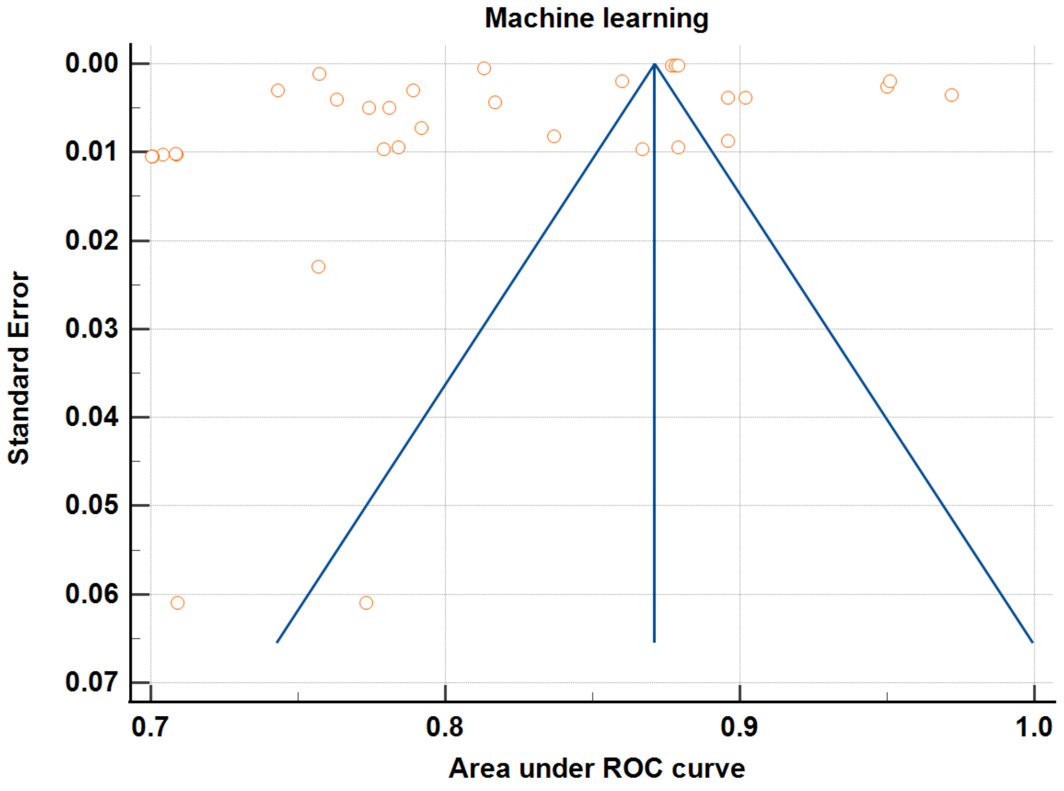

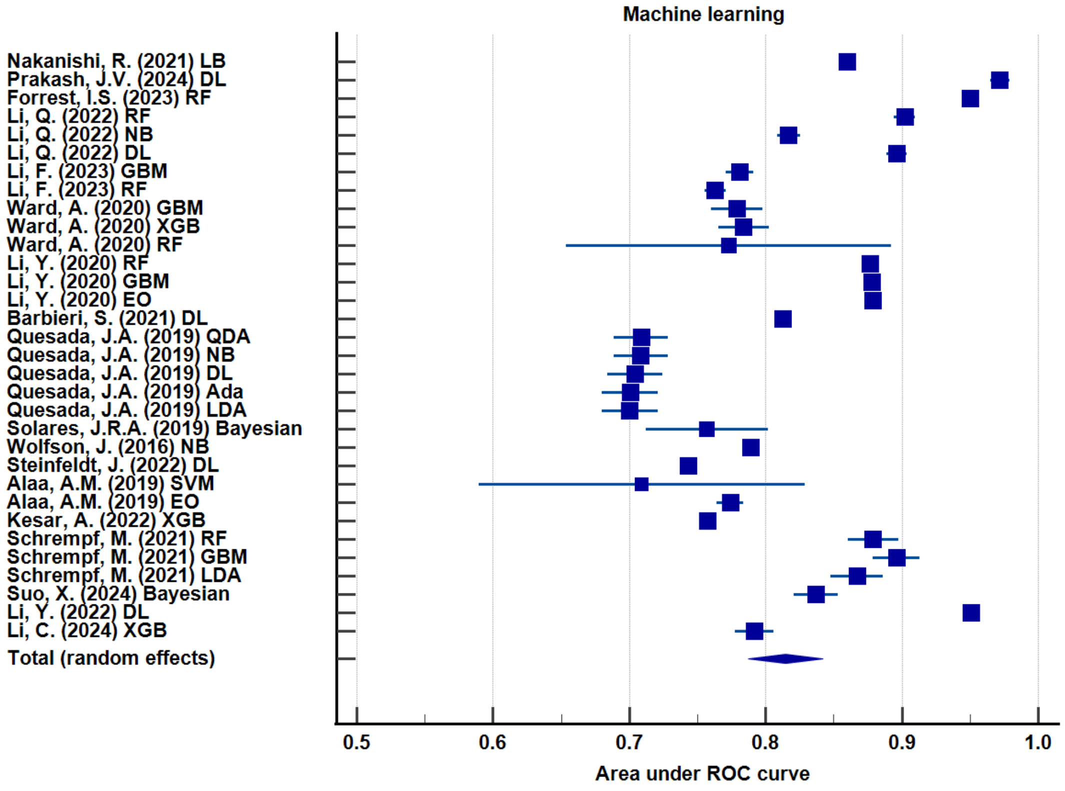

Logistic regression

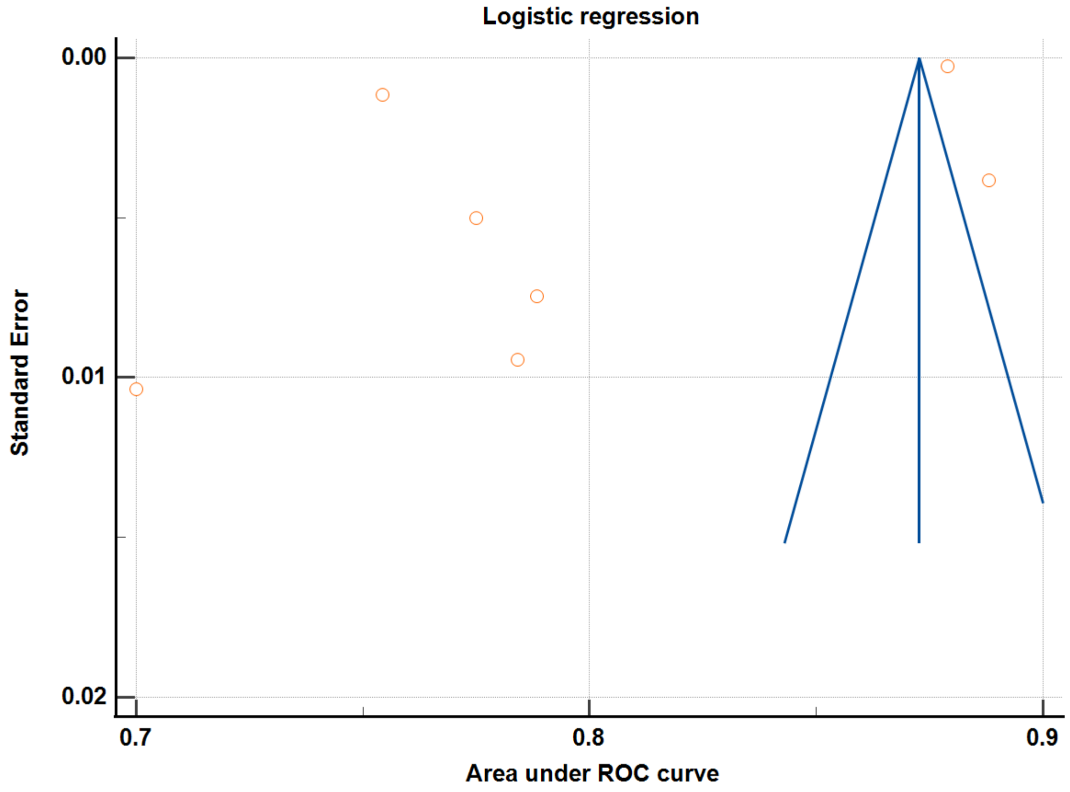

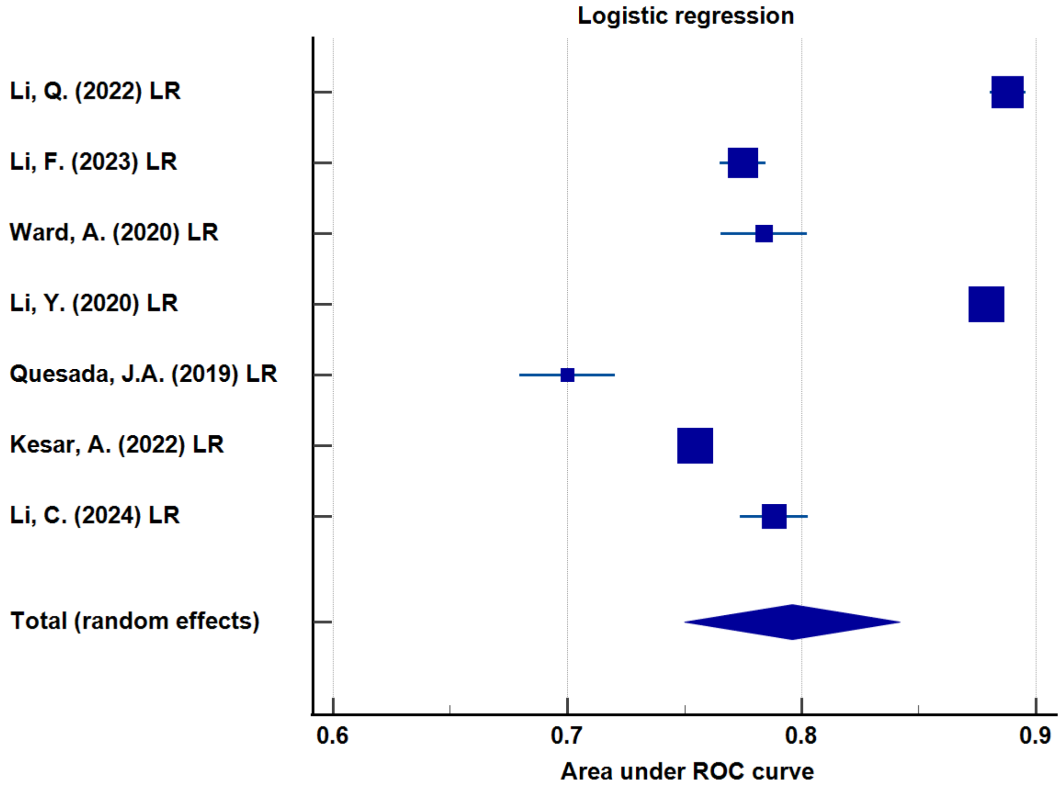

Cox

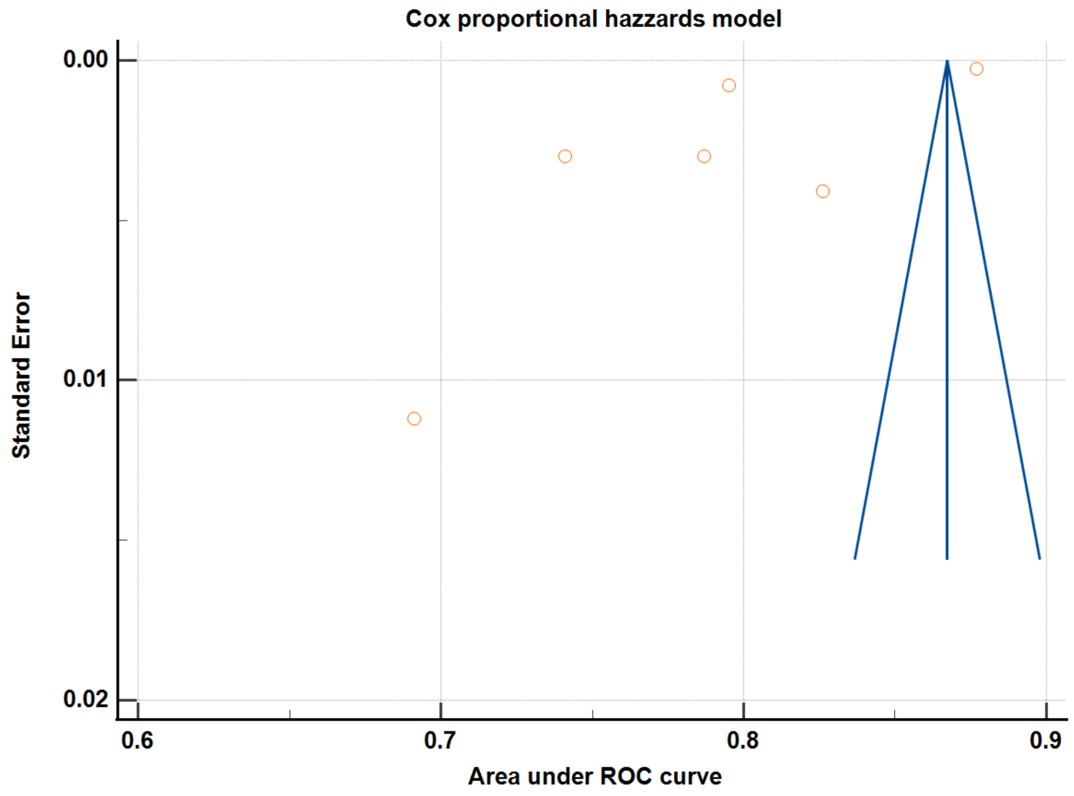
Cox proportional hazzards model
0.00
0.01
0.02
Standard Error
0.6
0.7
0.8
0.9
Area under ROC curve

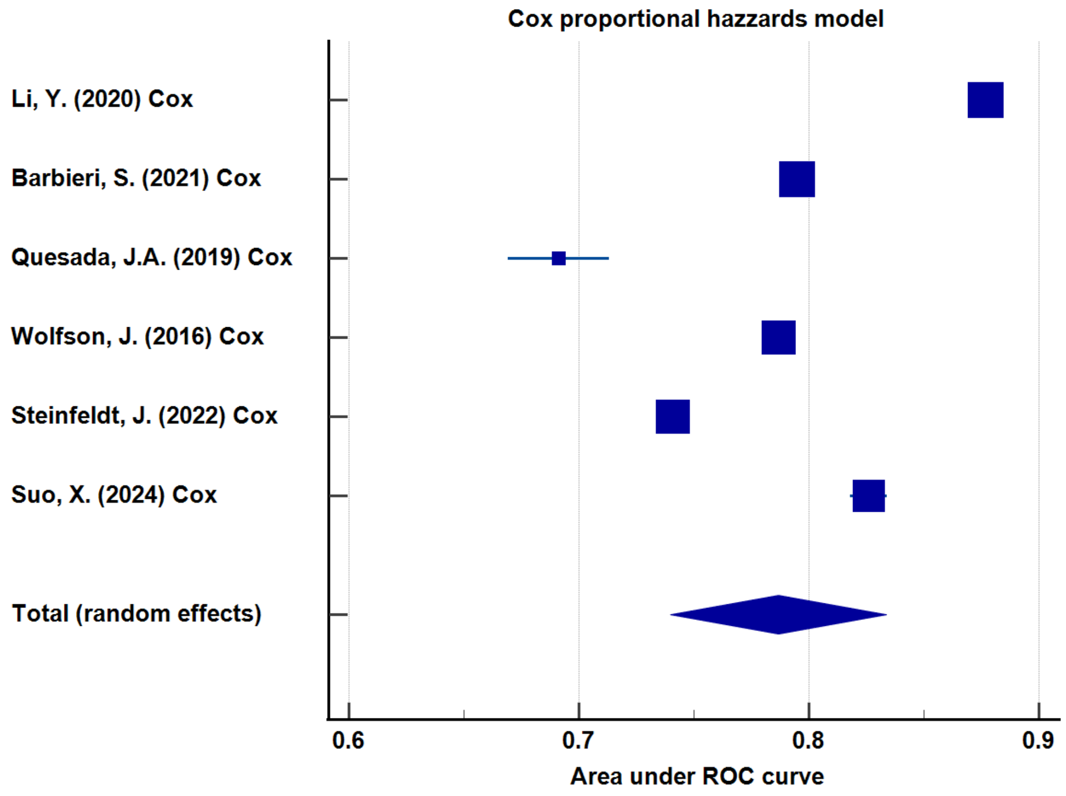
Cox proportional hazzards model
Li, Y. (2020) Cox
Barbieri, S. (2021) Cox
Quesada, J.A. (2019) Cox
Wolfson, J. (2016) Cox
Steinfeldt, J. (2022) Cox
Suo, X. (2024) Cox
Total (random effects)
0.6
0.7
0.8
0.9
Area under ROC curve

Pooled cohort equation

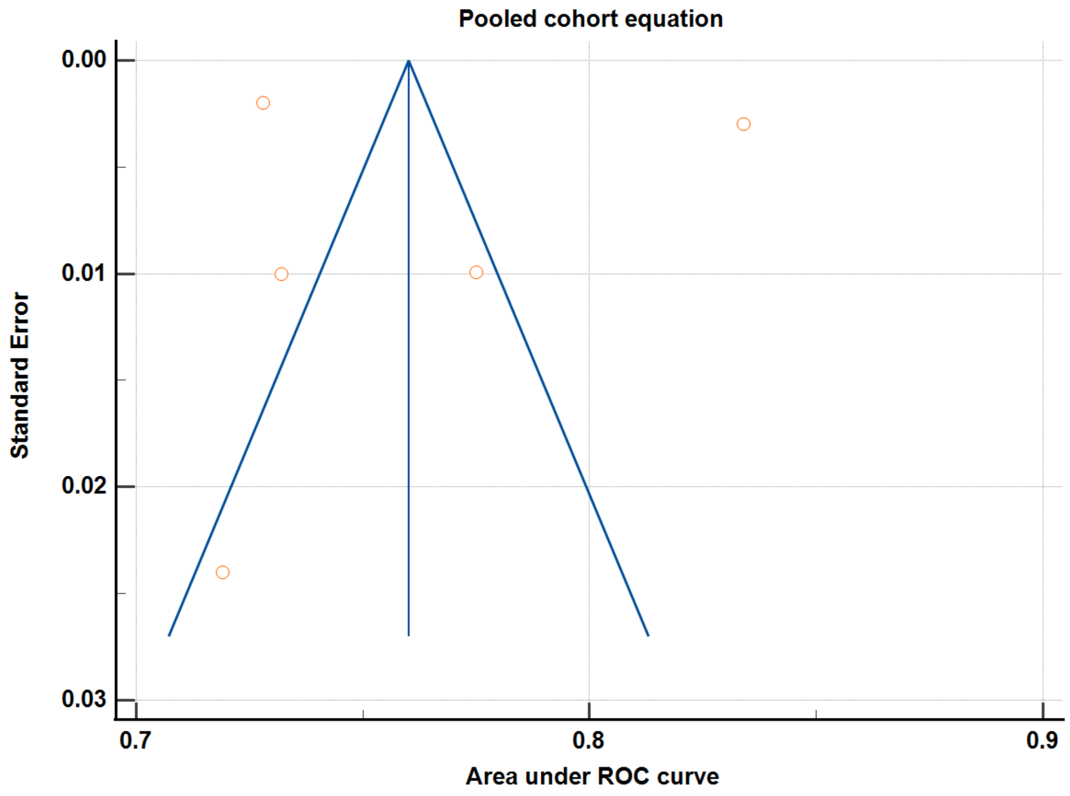

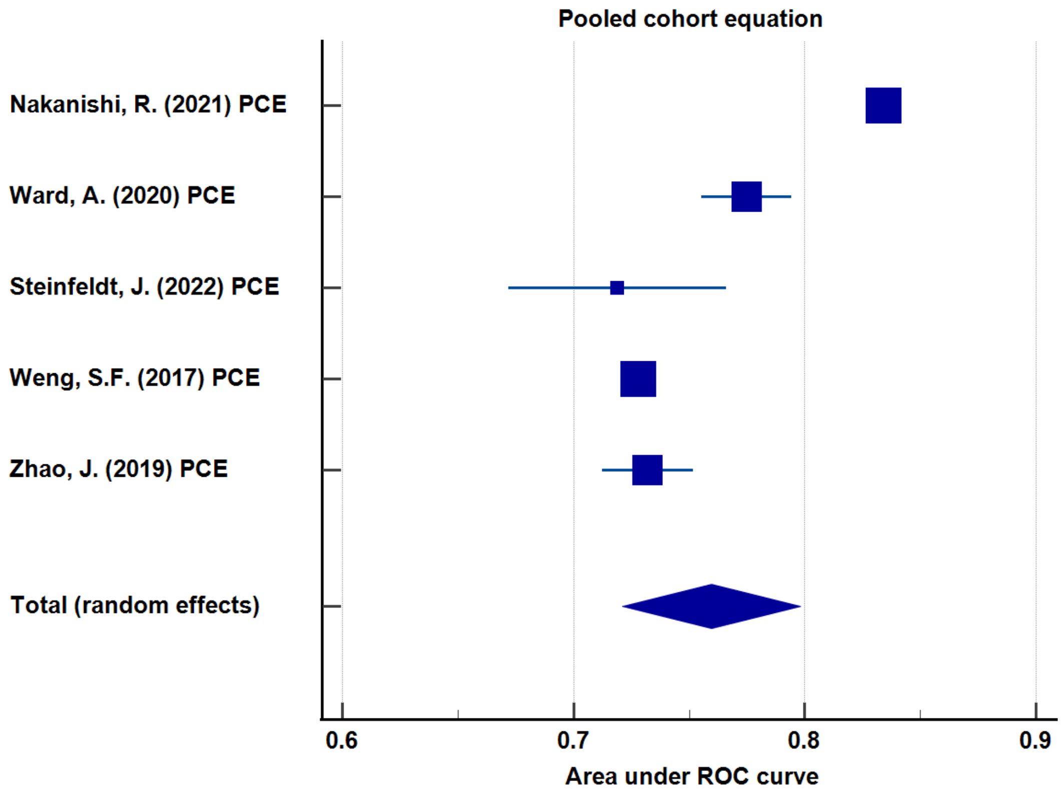

Qrisk

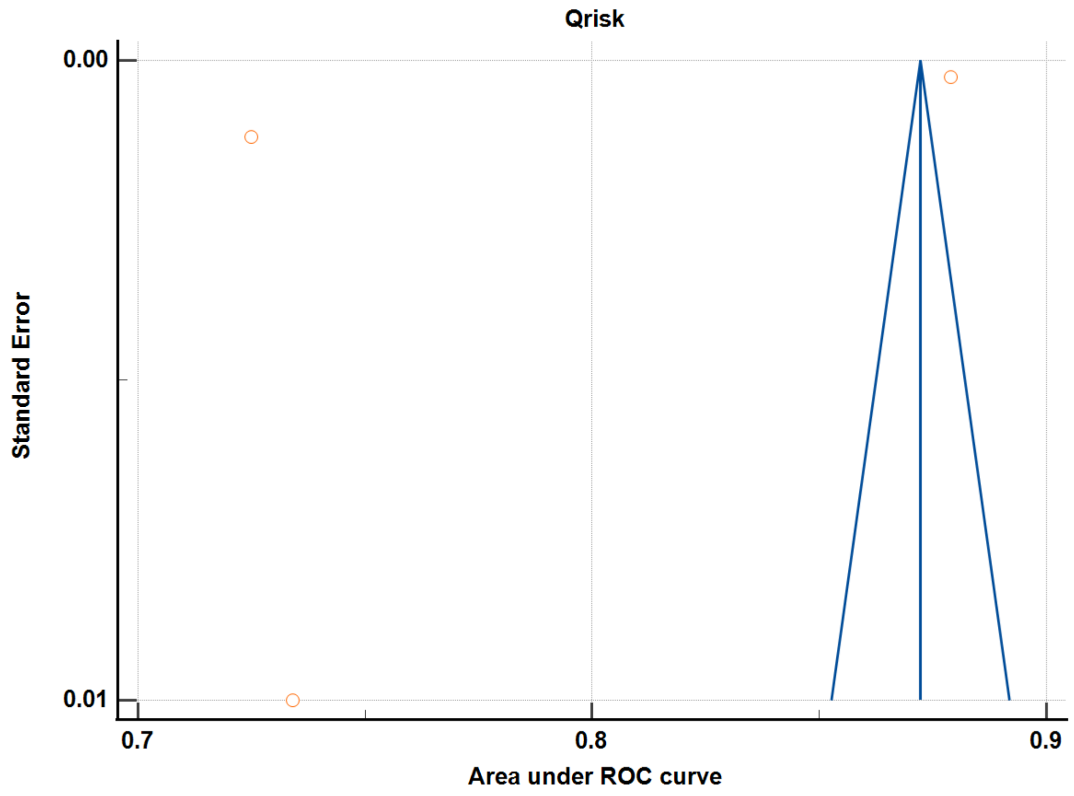
Qrisk
0.00
0.01
Standard Error
0.7
0.8
0.9
Area under ROC curve

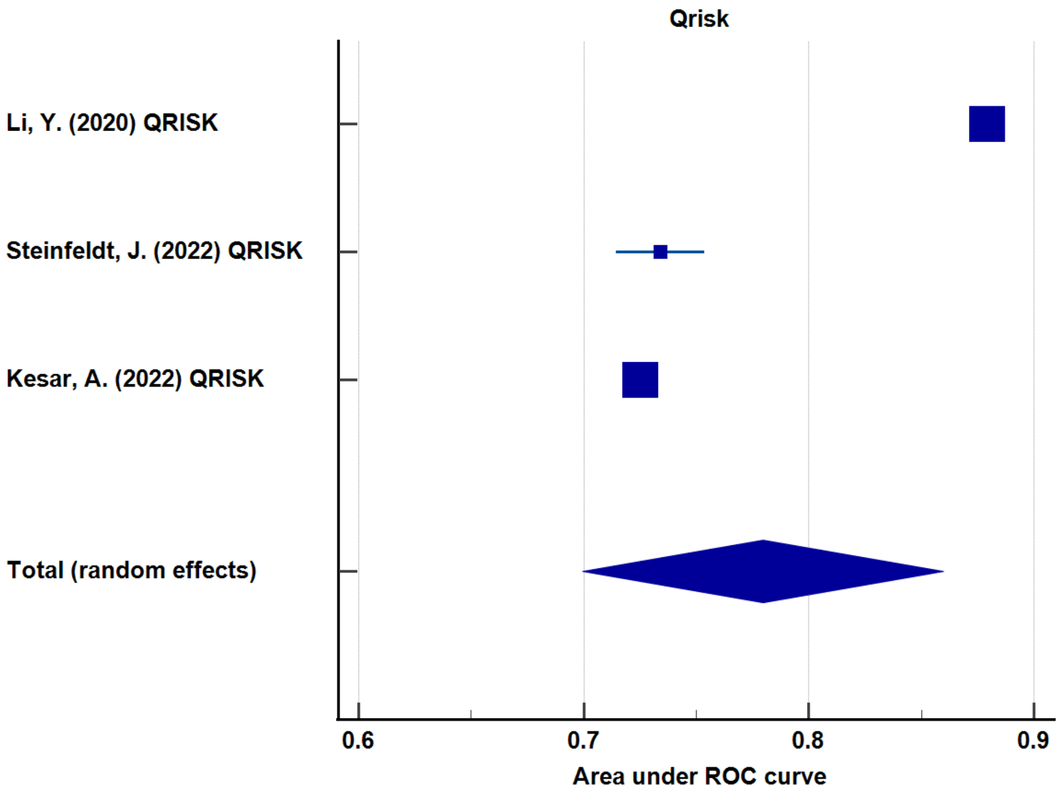
Qrisk
Li, Y. (2020) QRISK
Steinfeldt, J. (2022) QRISK
Kesar, A. (2022) QRISK
Total (random effects)
0.6
0.7
0.8
0.9
Area under ROC curve

Conventional

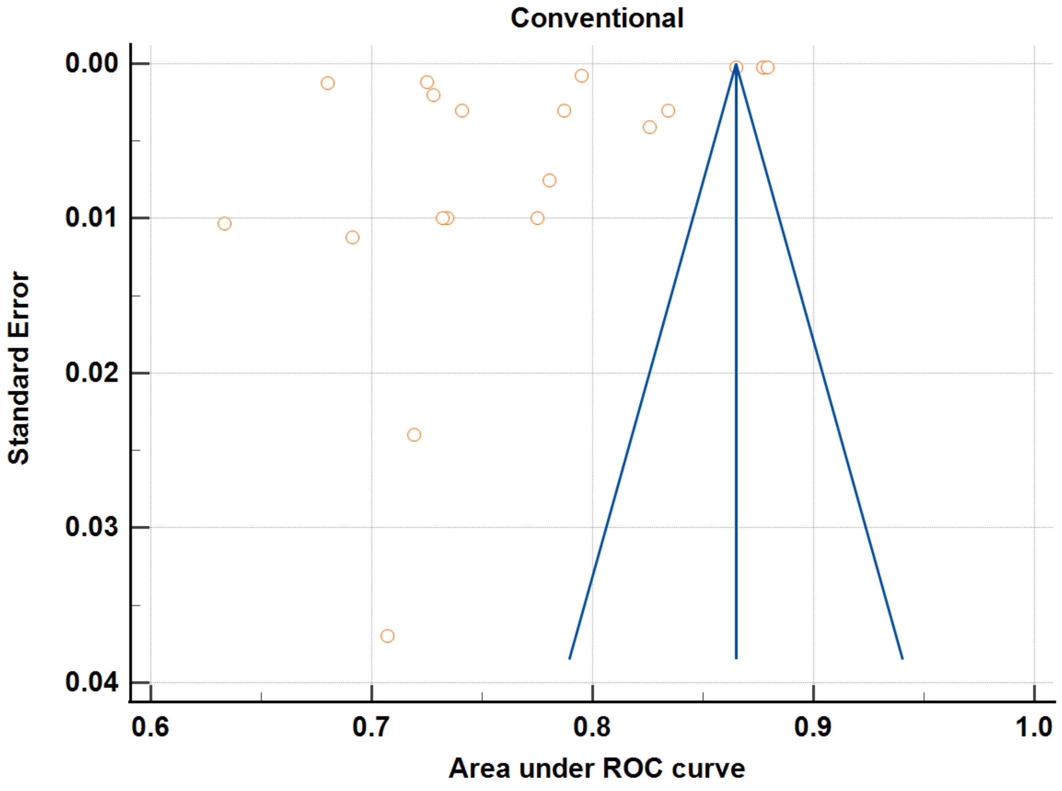

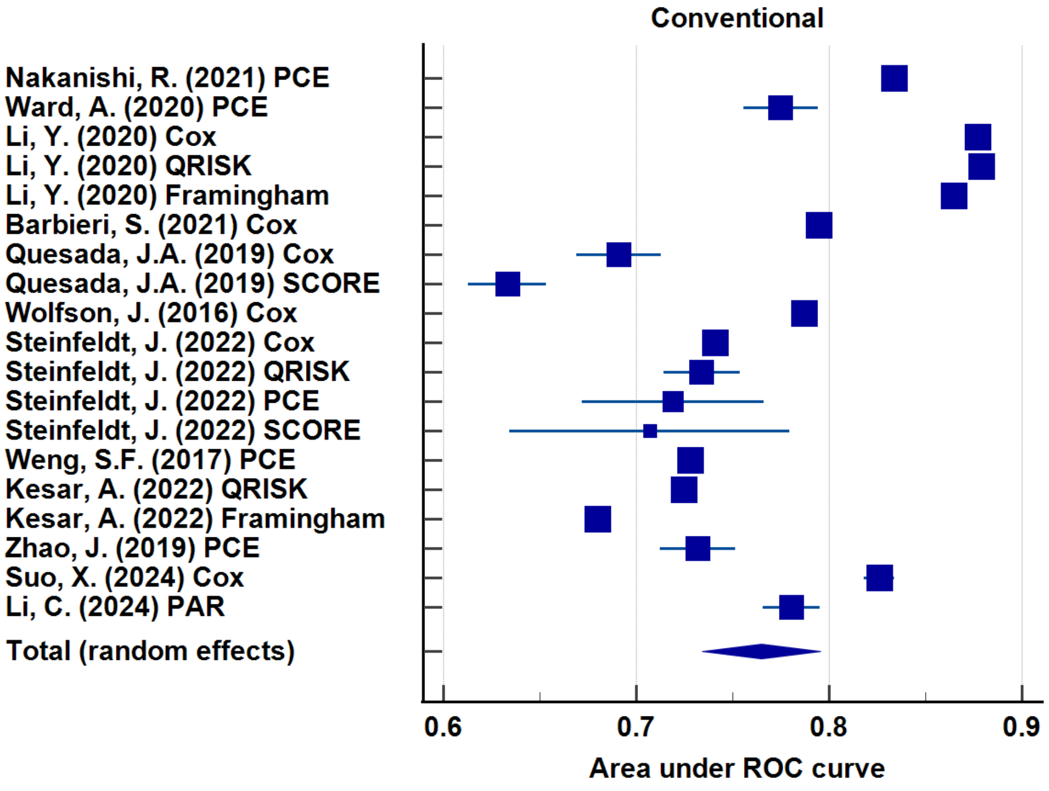

Supplement: ztae080_Supplementary_Data [file ztae080_supplementary_data.zip › Supplementary files S4.pdf]
