# Supplementary material for: Machine learning based prediction models for cardiovascular disease risk using electronic health records data: systematic review and meta-analysis
Source: Eur Heart J Digit Health. 2024 Oct 27;6(1):7–22. doi: 10.1093/ehjdh/ztae080 (PMC11750195; doi:10.1093/ehjdh/ztae080)
Supplement: ztae080_Supplementary_Data [file ztae080_supplementary_data.zip › Supplementary files S5.docx]

| **Domain** | **Key items** | **Explanation** |
| --- | --- | --- |
| 1. **Reference** | - 1. First Author | Name of authors. |
|  | - 1. Publication year | Published year of the study. |
|  | - 1. Published journal | Name of the journal in which the study was published. |
|  | - 1. Country/Region | Answer categories:   1. UK 2. USA 3. Europe 4. China 5. Others |
|  | - 1. Objective of the study | Answer categories:   1. Development of one or more new prediction model 2. Comparison across developed models 3. Comparison with existing modes 4. Validation of existing models |
| 1. **Data source** | 1. Source of Data | Answer categories:   1. CPRD 2. Biobank |
|  | 1. Data period | Key dates of collection and end of follow-up. |
|  | 1. Follow-up duration | Years of follow-up. |
|  | 1. Sample size | Sample size used for building the model. |
| 1. **Participants** | 1. Inclusion and exclusion criteria | Patients recruiting criteria. |
|  | 1. Settings | Answer categories:   1. Primary care/GP/outpatients 2. Secondary care/Hospitalization/inpatients |
|  | 1. Number of centres | Answer categories:   1. Single centre study 2. Multi-centre study |
| 1. **CVD outcomes** | 1. Clinical outcome | Answer categories:   1. CHD/IHD/CAD 2. Heart failure 3. Stroke/TIA 4. MI/Heart attack 5. PAD 6. CVD death 7. All-CVD not specific |
|  | 1. Was the outcome distribution unbalanced? | Answer categories:  Y - percentage of the larger class is more than 70%  N  NR - nothing is shown in figures, numbers, percentages |
|  | 1. Number of outcomes/events | The rate or probability of specific outcomes occurring within the dataset. |
| 1. **Features** | 1. Feature used before feature selection reported | Answer categories:  Y  N |
|  | 1. Feature used for algorithms reported | Answer categories:  Y  N |
|  | 1. Number of Predictors/Features | Number of features for building the model |
|  | 1. Type of predictors included | Answer categories:   1. Sociodemographic (age1, gender2, ethnicity3, SEP4) 2. Examination (BMI1, height2, weight3, SBP4, DBP5) 3. Laboratory result (Cholesterol1, glucose2) 4. Comorbidities (diabetic1, kidney2, hypertension3, hyperlipidemic4, metabolic5, Obesity6, Migraine7, RA8, SLE9, mental10, ED11, AF12) 5. Behavioural risk factors (smoking1, drinking2, physical inactivity3, diet4) 6. Family history 7. Drugs 8. Biomarkers 9. Imaging (ECG1, Echocardiographic2, CAC3) 10. Genetic 11. Risk score (ASCVD/PCE1, QRISK2, PRS3) |
| 1. **Missing data** | 1. Were there any missing values? | Answer categories:  Y  N  NR |
|  | 1. If there were missing values, were any variables removed from the dataset prior to the analysis because they had missing values? | Answer categories:  Y  N  NR |
|  | 1. Missing value methods | Answer categories:   1. Single imputation 2. Multiple imputation (MICE) 3. Complete case analysis (e.g., records with missing values were removed) 4. Missing values handled by the analytical method itself (e.g., decision tree, random forest) 5. No missing value |
| 1. **Model development** | 1. Machine Leaning models | Answer categories:   1. Deep learning 2. K-NN 3. Decision tree 4. Random forest 5. Naïve Bayes 6. Gradient boosting machine 7. Support vector machine 8. Other ensemble 9. Logistic 10. Cox 11. Others |
|  | 1. Baseline models | Answer categories:   1. Logistic regression 2. Cox regression 3. Itself different settings 4. QRISK 5. SCORE 6. Framingham 7. PCE 8. ASSIGN 9. China-PAR |
|  | 1. Pre-processing | Answer categories:   1. Cleaning 2. Normalization 3. Re-balance 4. Harmonisation 5. Sampling 6. De-identification methods 7. Quality check |
|  | 1. Were features selected prior to the actual analysis? | Answer categories:  Y  N  NR |
|  | 1. Feature selection methods | Answer categories:   1. Manually, based on expert knowledge, literature review 2. Data-driven methods (PCA1, LASSO2, Random Forest3, stepwiseLR4, chi25, Relief6) |
|  | 1. Hyperparameter selection method | Answer categories:   1. Grid search 2. Random search 3. Bayesian optimisation |
|  | 1. Ensemble techniques | Answer categories:   1. Bootstrap/Bagging 2. Boosting 3. Stacking |
| 1. **Model performance** | 1. Calibration | Answer categories:   1. Calibration plot (curve) 2. Calibration slope 3. Hosmer-Lemeshow test 4. Brier |
|  | 1. Discrimination | Answer categories:   1. AUC-ROC |
|  | 1. Classification | Answer categories:   1. PPV/Precision 2. NPV 3. TPR/Recall/Sensitivity 4. F1 score 5. Accuracy 6. Net reclassification improvement 7. TNR/Specificity 8. FPR 9. FNR 10. AUPRC |
|  | 1. Best performing model | The best performing model indicated in the study |
| 1. **Model evaluation** | 1. Internal validation | Answer categories:   1. Training-test splitting 2. Leave-one-out cross validation 3. N-fold cross validation 4. Bootstrapping |
|  | 1. External validation | Answer categories:   1. Temporal validation 2. Spatial validation |
|  | 1. Update | Answer categories:   1. Intercept recalibrated 2. Predictor effects adjusted 3. New predictors added |
|  | 1. Code availability | Answer categories:  Y  N |
|  | 1. Guidelines | Guidelines used |
